# A new human-based offensive defensive optimization algorithm for solving optimization problems

**DOI:** 10.1038/s41598-025-96559-6

**Published:** 2025-04-09

**Authors:** Ning Fang, Cheng Xu, Xuxiong Gong, Zhouhua Wu

**Affiliations:** 1 School of Electronic and Information Engineering, Beihang University, Beijing, 100191 China; 2 Wuhu Cigarette Factory of China Tobacco Anhui Industrial Co., Ltd., Anhui, 241000 Wuhu China; 3 Beijing Long March High Tech Co., Ltd., Beijing, 100176 China

**Keywords:** Global optimization, Metaheuristic algorithm, (CEC) 2017 benchmarks functions, Human-based, Applied mathematics, Computational science, Information technology

## Abstract

A novel human-inspired metaheuristic algorithm, termed Offensive Defensive Optimization, has been introduced to address single-objective optimization problems. This algorithm draws inspiration from the varied strategies utilized by players in board games, emulating and conceptualizing offensive and defensive behaviors within a hybrid search framework. The integration of mixed search behaviors facilitates a more efficient exploration and exploitation of the search space, thereby enhancing the algorithm’s capability to surmount local minima. The algorithm was evaluated using the benchmark test suites from the Congress on Evolutionary Computation (CEC) 2017 and 2022, in addition to two real-world engineering design problems. In comparison to eight well-established metaheuristic algorithms, the proposed method demonstrated superior performance in 80% of the CEC2017 cases and 72% of the CEC2022 cases, with statistically significant improvements. The results further indicate that the proposed algorithm exhibits satisfactory convergence efficiency, along with robust exploration and exploitation capabilities, while maintaining a balanced equilibrium between these two processes. Additionally, the outcomes of the engineering design problems suggest that the proposed algorithm effectively manages optimization tasks, demonstrating clear superiority and enhanced competitiveness.

## Introduction

Optimization problems are directed towards identifying appropriate system parameters to effectively fulfill the requirements and attain optimal outcomes. Despite the challenges posed by factors such as high-dimensional space and interdependence, stochastic optimization techniques, including evolution and metaheuristic optimization algorithms^1,2^ , facilitate the resolution of optimization problems with greater ease and feasibility. By employing search strategies characterized by randomness, this technology enables the discovery of nearly optimal solutions, while minimizing the need to traverse intricate solution spaces^3–11^. The objective of these optimization algorithms is to employ stochastic processes and group search strategies to discover algorithms that closely approximate the optimal solution within a reasonable computational timeframe. These algorithms do not rely on specific problem domains and can be applied across various fields, such as hand biometric recognition^12^, prediction of byproducts for water treatment plants^13^, antenna design^14^, maximum power point tracking problems for photovoltaic systems under local shadow conditions^15^, optimization of cognitive femtocell networks^16^, optimization of multilayer absorbers^17^, and time cost trade-off problems^18^. Optimization algorithms and numerous applications mutually drive the development of increasingly innovative techniques, including the emergence of hybrid methods^19–21^.

The metaheuristic algorithm is renowned for its utilization in a random-search strategy. The incorporation of stochastic elements within a designated search process facilitates problem-solving, lending an air of enchantment to the algorithm’s capabilities. Since the inception of traditional metaheuristic algorithms, numerous approaches for addressing global optimization problems have emerged, drawing inspiration from animal behavior, plant attributes, natural phenomena, human conduct, and other sources. These methodologies have gained significant attention in recent times. Pavel Trojovský et al. introduced the Pelican Optimization Algorithm (POA) to simulate the hunting behavior of pelicans in animal behavior^22^. Similarly, Mohapatra et al. presented the American zebra optimization algorithm (AZOA), which emulates the social behavior observed in American zebras in their natural habitat. Notably, American zebras exhibit distinctive social characteristics and undergo leadership training, enabling them to disassociate from their familial group and join a separate group prior to reaching adulthood. The promotion of diversity is facilitated by the departure of the baby zebra, as it prevents intra-familial mating. Furthermore, leadership training of American zebras plays a crucial role in ensuring this convergence, as it governs the group’s speed and direction. The indigenous social lifestyle behavior exhibited by American zebras serves as a primary source of inspiration for the proposed AZOA metaheuristic^23^. Dehghani et al. developed Green Anaconda Optimization (GAO), which emulates the natural behavior of anacondas in their work^24^. The underlying motivation behind the development of GAO stems from the observation of the male species’ ability to locate female species during the mating season, as well as the hunting tactics employed by forest pythons. The mathematical modeling of GAO is grounded in the simulation of these two strategies, specifically in the exploration and exploitation phases, similar to those observed in forest pythons. In the realm of simulating natural phenomena, Kaiping Luo introduced the water flow optimization (WFO) algorithm, drawing inspiration from the fluid dynamics exhibited by water in its natural environment^25^. The presented optimizer models the hydraulic process of water particles descending from elevated areas to lower areas by employing two mechanisms: (1) laminar flow and (2) turbulence. Mirrashid et al. introduced the Transit Search (TS) approach, which relies on a widely recognized method for detecting exoplanets^26^. Su et al. proposed an optimization algorithm known as the rime or rime optimization algorithm, which is inspired by the physical phenomenon of rime ice^27^. The RIME algorithm emulates the growth dynamics of soft and hard haze within haze ice, formulates a search strategy for soft haze and a puncture mechanism for hard haze, and accomplishes exploration and exploitation behavior using optimization methods. In addition, the algorithm enhances the greedy selection mechanism and updates the population during the optimal solution selection stage, thereby augmenting the utilization capability of RIME. Dalirinia et al. introduced the lotus effect optimization algorithm (LEA) to simulate plant characteristics, specifically the movement process of dragonflies during flower pollination and the self-cleaning lotus leaf effect of water on flowers and leaves^28^. Drawing inspiration from fruit tree horticulture, Kaveh et al. simulated behaviors such as irrigation, fertilization, pruning, and grafting, and incorporated operators such as annual growth, screening, and grafting to develop the Orchard Algorithm (OA)^29^. To simulate human behavior, Malik Braik et al. introduced the Alibaba and Forty Thieves (AFT) algorithm, wherein the strategies employed by forty thieves in their quest for Alibaba were translated into mathematical models, thereby facilitating the process of exploration and exploitation^30^. Similarly, Bing Ma et al. proposed the running city game optimizer (RCGO), which emulates the actions of game participants engaged in playing Run City games^31^. The mathematical establishment of the RCGO is attributed to the utilization of three novel search strategies: siege, defense, and elimination selection. Zhang et al. introduced the Special Forces Algorithm (SFA), an optimization algorithm inspired by human behavior, as a means to achieve this^32^. In general, researchers exhibit a strong preference for newly developed algorithms that emulate animal or human behavior.

Irrespective of the foundational inspiration for a particular optimization algorithm, the explicit behavioral rules governing particles within populations, along with their collective strategic behaviors, constitute essential elements for achieving significant advancements. Extracting specific insights from long-term patterns of human behavior and translating these insights into mathematical models can infuse metaheuristic algorithms with renewed vigor for addressing optimization challenges. Chessboard games represent the outcome of human-engineered simulated behavior and decision-making, developed after extensive contemplation. The rules governing the movement of chess pieces on the board are relatively straightforward, yet players engage in dynamic behavioral processes during the game, guided by these simple rules, with a singular and well-defined ultimate objective. This process bears remarkable resemblance to metaheuristic optimization algorithms, encouraging the development of more effective optimization algorithms inspired by board games.

This study draws inspiration from board games and constructs a mathematical model of search behavior by incorporating the chess process of chess games. The proposed approach, referred to as Offensive Defensive Optimization (ODO), leverages offensive behavior to explore the global problem solution space and defensive behavior to explore the local problem solution space. By effectively balancing exploration and exploitation, this design mitigates the risk of being trapped in local minima, thereby enhancing the optimization capability of ODO. The performance of ODO was evaluated using two rigorous benchmarks derived from the Evolutionary Computing Conference (CEC), namely CEC2017 and CEC2022. To ascertain its optimization capability, ODO was compared with a set of 12 random optimization techniques. The comparison encompassed a selection of recently published and analogous algorithms. To assess its efficacy in practical engineering applications, the pressure vessel design and gas transmission compressor problems were employed as test cases. Based on the analysis of convergence curves and statistical data, ODO has emerged as the most promising optimizer among its competitors. The main contributions of this study are as follows:

- A novel metaheuristic algorithm, termed as Offensive Defensive Optimization, has been proposed. This algorithm ingeniously transmutes various offensive and defensive strategies inherent in gameplay into search behaviors via mathematical modeling. By striking a balance between exploration and exploitation, the optimization capability of the algorithm is significantly enhanced.
- A novel hybrid search behavior framework has been developed, integrating various offensive and defensive behaviors. This integration significantly bolsters the optimization algorithm’s capacity to escape local minima. The amalgamation of search behaviors facilitates a more effective exploration and exploitation of the search space, thereby optimizing the algorithm’s performance.
- ODO was assessed by employing two demanding CEC benchmarks and two practical engineering problems to ascertain its proficiency in addressing a wide range of optimization problems with diverse characteristics. The experimental findings demonstrate that ODO outperforms other comparable optimizers.

The subsequent section of this article is organized as follows: A comprehensive overview of recently published algorithms that have inspired the ODO algorithm is presented in section “Related work”. And in section “Offensive defensive optimization”, it is provided that a thorough introduction to the ODO algorithm, encompassing its sources of inspiration, mathematical model, and flowchart. Then, the results and discussion comes up in section “Calculation and analysis”, which entail the testing of the new algorithm on a test set comprising 41 test programs. By employing an extensive test set encompassing all the genuine parameter single-objective optimization test sets from CEC2017 and CEC2022, the validation of the algorithms is enhanced as it mitigates the risk of overfitting to some degree, in contrast to utilizing a limited number of test sets. Finally, section “Conclusion” presents conclusive remarks and offers recommendations for future research.

## Related work

### Human-based metaheuristic algorithms

Among the plethora of optimization algorithms, the approach of simulating human behavior is prominent. Numerous researchers have successfully translated human behavior into mathematical models to address optimization problems, yielding highly satisfactory outcomes. These optimization algorithms offer valuable insights for the development of novel algorithms. Several optimization algorithms proposed in recent years are grounded in the emulation of human behavior.

Education has played a pivotal role in fostering ongoing advancement and societal progress, thereby finding applications in optimization algorithms. Numerous optimization algorithms that emulate human learning patterns have been introduced. One such algorithm, known as teaching-learning-based optimization (TLBO), is a potent and effective optimization technique^33^. Its fundamental concept draws inspiration from the influence exerted by a teacher’s efforts on students, specifically how the teaching proficiency of an instructor impacts their pupils’ academic achievements. This algorithm encompasses a two-stage optimization process, namely, the teacher and learning stages, which facilitates students to acquire knowledge from both their instructor and peers. To enhance the overall optimization performance of TLBO, Akbari et al. introduced an upgraded variant known as Teaching-Learning-Studying-Based Optimization (TLSBO)^34^ . The suggested enhancement entails the incorporation of a novel learning strategy into TLBO, wherein each participant leverages information from a randomly chosen individual to refine its own position. In addition to the aforementioned contributions, Wu et al. presented an enhanced methodology utilizing the TLBO algorithm, incorporating the Q-learning technique in reinforcement learning (RL) and implementing a switching mechanism between two distinct learning modes during the learner stage^35^. Furthermore, Das et al. introduced the Student Psychology-based Optimization (SPBO) algorithm, which draws inspiration from the behavior of students who strive to exert greater effort to enhance their academic performance and attain the status of the top student in their class^36^. Zhang et al. introduced the group teaching optimization algorithm (GTOA), which draws inspiration from the group teaching mechanism commonly employed in educational settings^37^. This approach initially involves dividing students into distinct groups based on predetermined criteria and subsequently employing tailored teaching strategies to enhance the knowledge of each group. Nevertheless, the GTOA may encounter challenges in addressing complex optimization problems, as it may become confined to local optima owing to the limited communication between the exceptional and average groups. Based on GTOA, Zhang et al. introduced a novel iteration, referred to as the group teaching optimization algorithm with information sharing (ISGTOA), with the aim of enhancing its performance^38^. Similar to its predecessor, ISGTOA avoids the inclusion of additional control parameters and promotes communication between exceptional and ordinary groups by reutilizing individuals from the two established profiles. In practical settings, individuals acquire knowledge from their immediate environment akin to students, resulting in diverse perspectives on learning and learning approaches. This is also a contributing factor to the extensive size of the family of learning-behavior optimization algorithms.

In addition to formal education, certain forms of specialized skill acquisition and training serve as valuable sources of inspiration for enhancing algorithm optimization. A notable instance is the driving training-based optimization (DTBO) algorithm introduced by Dehghani et al., which emulates the practices involved in human driving training^39^. The underlying motivation driving the development of the DTBO stems from the pedagogical methods employed in driving schools and the instructional techniques employed by driving instructors. The optimization process encompasses multiple stages, which involve the training of driving instructors, cultivation of students through teacher skills, and practical applications. Trojovská et al. introduced the Chef-based Optimization Algorithm (CBOA), which draws inspiration from the acquisition of culinary expertise through training. The CBOA algorithm employs a mathematical representation of each stage of the cooking training process, positing that simulating this process can enhance both the global search capability for exploration and local search capability for exploitation^40^. Trojovský et al. introduced Language Education Optimization (LEO) as a proposed approach for addressing optimization problems^41^. LEO draws inspiration from the pedagogical process of foreign language education, wherein language instructors impart language skills and rule training to students within language schools. The mathematical formulation of LEO encompasses three distinct stages: (i) selection of teachers by students, (ii) collaborative learning among students, and (iii) individualized practice. Givi et al. introduced a Skill Optimization Algorithm (SOA) as a solution to optimization problems^42^. The conceptual basis for the design of SOA is derived from human endeavors to acquire and enhance skills. The mathematical representation of the different SOA phases encompasses two stages: (i) exploration, which involves acquiring skills from experts, and (ii) skill improvement through practice and personal effort.

Human capacity for adaptability is a formidable trait that facilitates swift adjustment to novel surroundings and circumstances. This adaptability is not inherent but rather derived from accumulated experiences and acquired wisdom. When confronted with unfamiliar environments and phenomena, individuals consistently demonstrate the ability to discern their survival strategies based on past encounters and observed patterns. The capacity to acquire and synthesize information is a significant attribute that distinguishes humans from other organisms. We possess the capability to rapidly assimilate novel knowledge and skills as well as derive overarching principles and guidelines from this acquired knowledge and skillset. This aptitude for learning and summarizing not only enhances our adaptability to unfamiliar surroundings and phenomena but also stimulates the development of novel optimization algorithms.

According to Mirrashid et al., the human mind is a multifaceted biological entity with distinct capabilities^43^. It is capable of being educated, and its reasoning processes can evolve based on the acquired knowledge. From a psychological standpoint, what individuals do not currently comprehend may be deemed illogical. However, this perception can shift over time. The concept of “incomprehensible yet comprehensible temporal logic” (IbI) , which is currently considered non-logical, has the potential to evolve into a recognized form of logic in the future. By transforming the concept of IbI into a mathematical model, Mirrashid et al. developed a new optimization algorithm called ILA.

The migration algorithm (MA) was introduced by Pavel Trojovský et al. with the objective of enhancing work, education, economy, and living conditions, drawing inspiration from the human migration process^44^. The mathematical modeling of MA is structured into two stages, allowing for the investigation and application of the proposed method throughout the search process. In the exploration phase, the algorithm population was updated by simulating the selection of a migration destination from a range of available options. During the exploitation stage, the algorithm population undergoes updates contingent upon the endeavors of individuals within the migration area, as they strive to acclimate to the novel environment and enhance their respective circumstances. Kamran Zolfi introduced the Gold Rush Optimizer (GRO), which incorporates three fundamental principles of gold exploration, namely migration, collaboration, and elimination, to emulate the search for gold by miners during the historical gold rush period^45^.

The Past Present Future (PPF) optimization algorithm was proposed by Naik et al., drawing inspiration from the societal phenomenon of individuals learning from successful counterparts^46^. PPF operates on the premise that an individual’s future prospects are contingent on their past experiences and ongoing efforts. Furthermore, the enhancement of one’s future life is influenced by successful individuals. This algorithm employs a strategy of partitioning the population into subpopulations, which then utilizes a switching mechanism to effectively monitor alterations in the optimal positions of individuals.

Current metaheuristic algorithms inspired by human behavior continue to encounter challenges related to the mechanisms of behavior coupling and the thorough exploration of solution spaces. When drawing inspiration from human behavior, it is essential for researchers to distill these behaviors into fundamental, easily executable, and logically independent actions. Concurrently, they must develop behavioral frameworks that facilitate optimization by enabling nonlinear coupling among these basic actions. This approach aims to prevent premature convergence and maximize species diversity, thereby effectively navigating complex solution spaces. These principles are exemplified in human-invented chess games, where the movement rules of chess pieces represent basic behaviors characterized by simplicity and logical independence. The player’s strategic selection of chess pieces during each move serves as a behavioral framework. Moreover, players frequently utilize every area of the chessboard, demonstrating effective exploration of intricate solution spaces. These insights encourage the development of more advanced optimization algorithms inspired by board games.

### Motivation

Throughout the ongoing evolution of human civilization, diverse forms of confrontational communication have consistently emerged, skillfully distilled into a variety of engaging and intellectually stimulating board games. These games, such as Chinese Chess, necessitate participants to strategically position their pieces on the game board to secure the most advantageous position, with the ultimate goal of winning the game. Each move in a chess game is contingent upon the player’s observation of the board’s state and subsequent decision-making, taking into account the situation of both opponents. Whether in offense or defense, chess players maneuver different pieces for varied objectives. Particularly in offense, different pieces pose distinct threats and occupy unique positions, leading to a diverse range of game scenarios. The choice of mobile pieces by each player is also subject to the prevailing situation and their individual capabilities. The duration of a chess game can vary, but it invariably culminates in a decisive outcome.

The primary appeal of board games resides in the necessity for both participants to strategically employ a limited set of pieces, maximizing the utility of each within the fewest possible moves to achieve victory. This concept holds significant relevance for metaheuristic algorithms. The behavior and actions of particles within a population must be neither singular nor excessively numerous, analogous to the variety of chess pieces. In most instances, individual chess pieces do not secure victory merely by executing their designated functions; however, they can achieve success with a single decisive move during critical moments. This phenomenon parallels the behavior of particles in optimization processes, where they may unexpectedly converge on or discover the optimal solution following initial exploratory movements. Consequently, we aim to develop a motion framework and behavioral pattern conducive to particle optimization, guided by this adversarial thinking, to attain the optimal solution.

### General description of optimization problems

Optimization refers to the systematic procedure of identifying the most favorable solution from a range of possibilities in an optimization problem guided by specific criteria. Optimization encompasses the resolution of optimization problems, wherein the objective may involve either maximizing or minimizing certain processes. In this context, if *S* denotes the search space, *F* and $F\in S$ represent the collection of acceptable solutions for *S*, and *f* signifies the objective function or fitness, the act of minimizing entails locating $X\in F$ in Eq. (1), which delineates the definitions of minimization and maximization.

1 $$\begin{aligned} f({{X}^{*}})\le f(X)\, \forall X\in F \end{aligned}$$

The group optimization technology employs a random generation process to create a candidate solution ${{X}_{i}}$, $i=1,2,\ldots ,N$, within the limited search space *S*, defined by lower bound *LB* and upper bound *UB*. *N* Individuals are then guided to move within this search space based on specific rules. The movement ceases once a predetermined number of iterations or convergence conditions are met. Equation (2) is used to determine the global best solution ${{X}_{gb}}$ from the population for each movement.

2 $$\begin{aligned} {{X}_{gb}}=\min \{f({{X}_{i}})\} \end{aligned}$$

In general, the maximization problem can be converted into a minimization problem by employing Eq. (3). Consequently, subsequent discussions have focused on minimizing this problem.

3 $$\begin{aligned} {{X}_{gb}}=\max \{f({{X}_{i}})\}=\min \{-f({{X}_{i}})\} \end{aligned}$$

When addressing constrained optimization problems, metaheuristic algorithms incorporate a penalty factor, denoted as $\lambda$, to incorporate constraints into the objective function, as demonstrated in Eqs. (4)–(6): In these equations, *C*(*X*)represents the constraint value, *g*(*X*) denotes the inequality constraint, and *q* signifies the total number of constraints. Specifically, when the constraint conditions were satisfied, the value of $\lambda$ was set to zero. Conversely, when the constraint conditions are not fulfilled, $\lambda$ assumes substantial value, specifically ${{10}^{20}}$, as stated in this article.

4 $$\begin{aligned} {{F}_{fitness}}(X)&=f(X)+\lambda \sum \limits _{i=1}^{q}{C_{i}^{2}(X)} \end{aligned}$$

5 $$\begin{aligned} {{C}_{i}}(X)&=\max \left( 0,{{g}_{i}}(X) \right) \end{aligned}$$

6 $$\begin{aligned} {{g}_{i}}(X)&\le 0\, , \, i=1,2,\ldots ,q \end{aligned}$$

In a comprehensive manner, metaheuristic optimization algorithms aim to minimize ${{F}_{fitness}}$. To simplify matters, the subsequent sections consistently employ the symbol *f* to denote the objective function or fitness value.

## Offensive defensive optimization

The competitive dynamics of chess games can be conceptualized as a process of solving optimization problems. Each move or placement of a player’s chess piece is aimed at identifying the most advantageous position or scenario for victory, thereby forming an optimization algorithm referred to as game process optimization. In the context of the Offensive Defensive Optimization (ODO) algorithm, there are *N* players, each denoted as ${{X}_{i}}$, $i=1,2,\ldots ,N$. Each player strives to secure the most favorable position in the game space to win, i.e., to find the optimal solution ${{X}^{*}}$ in the solution space of the problem. Assuming the optimization problem has *D* dimensions, the player will persistently explore positions superior to the current global optimal solution ${{X}_{gb}}$ in each dimension $x_{i}^{j}$, $j=1,2,\ldots ,D$, employing both offensive and defensive behaviors. Upon iteration, the current global optimal solution ${{X}_{gb}}$ becomes the optimal solution $f({{X}_{gb}})$ identified by the ODO algorithm for the problem.

Board games are typically competitive games performed by two players, necessitating the implementation of two distinct strategies: offensive and defensive. The attainment of victory in chess games relies on intricate dynamics encompassing elements such as attack and defense, virtual and tangible aspects, and global and localized alterations. These two strategies are also present in the ODO algorithm, wherein the offensive approach entails launching attacks from various dimensions and employing diverse chess pieces to target specific dimensions. The defensive strategy diverges from a comprehensive offensive approach, as it is the defensive side that determines which dimensions remain unaltered and which dimensions employ defensive movement strategies.

### Offensive strategy

In the context of a board game, the movement of each piece has two objectives. First, it aims to advance the piece towards the adversary’s encampment, with the intention of launching an attack on their vital commander. Second, it endeavors to establish a strategically advantageous position that poses a threat to the player, thereby disrupting their formation. Hence, we incorporate the positional attribute $P_{i}^{j}$ of every piece movement and the corresponding threat scenario $T_{i}^{j}$ into the optimization problem, which involves determining the numerical alteration $x_{new}^{j}$ for each dimension *j*, as depicted in Eq. (7), through iterative processes. The objective was to identify the optimal solution and represent the resultant attack outcome using $X_{new}^{a}=(x_{new}^{1},x_{new}^{2},\ldots ,x_{new}^{D})$.

7 $$\begin{aligned} x_{new}^{j}=P_{i}^{j}+T_{i}^{j} \end{aligned}$$

Within the scope of offensive strategy, a player is endowed with four unique modes of movement. Each of these modes will be elaborated upon in detail in the ensuing discussion.

#### Distributed offensive threat-I

In the context of chess games, the strategic placement of pieces in pivotal positions on the board is a prevalent offensive tactic. The more troops are deployed to effectively divert and engage the adversary’s attention across various locations, the greater the opportunities for victory when focusing the remainder of their forces at a specific point to launch an attack. This strategy finds relevance in optimization problems as well. For an undetermined problem solution space, its spatial distribution characteristics can vary significantly, complicating the identification of crucial positions. Nevertheless, in optimization, the development of useful spaces as extensively as possible is instrumental in obtaining the problem’s optimal solution. Considering that the positions of each individual in the population are fundamentally distinct, we employ the global optimal solution, the positions of random individuals, and the currently updated individuals to cultivate new spaces. This behavior, aimed at developing an expansive problem solution space, is termed as Distributed Offensive Threat-I.

In this offensive threat, the moving position $P_{i,dI}^{j}$ is affected by three positions, and for each dimension, the new position is simulated using Eq. (8).

8 $$\begin{aligned} P_{i,dI}^{j}=(x_{gb}^{j}+x_{r}^{j}+x_{i}^{j})/3 \end{aligned}$$

where ${{X}_{r}}$denotes a randomly chosen individual from the population. ${{X}_{i}}$ represents the i-th individual.

The simulated attack threat resulting from movement $P_{i,dI}^{j}$is represented by Eq. (9).

9 $$\begin{aligned} T_{i,dI}^{j}=h\times (P_{i}^{j}-x_{i}^{j}) \end{aligned}$$

where variable *h* is a random number that signifies the level of peril imposed upon the adversary by each maneuver, as determined by the mathematical expression denoted by Eq. (10).

10 $$\begin{aligned} h=2\sin \left( \frac{\pi }{2}n \right) \end{aligned}$$

where *n* is a uniformly distributed random number within the interval [−1,1].

#### Concentrated offensive threat-I

In challenging scenarios of a board game, the combatants, on one hand, endeavor to swiftly mobilize their dispersed forces across multiple paths into the battlefield, posing a dispersed threat to the adversary. On the other hand, both factions are striving to concentrate their forces on the adversary’s critical areas, aiming to secure a decisive victory through concentrated attack. This centralized offensive strategy is mirrored in the process of population optimization. Here, the movement range of individuals within the population is constrained; they cannot deviate significantly from their current position and must remain relatively unaffected by external factors. Consequently, we employ the global optimal solution and the position of the currently updated individual to ascertain the new position $P_{i,cI}^{j}$, as depicted in Eq. (11).

11 $$\begin{aligned} P_{i,cI}^{j}=(x_{gb}^{j}+x_{i}^{j})/2 \end{aligned}$$

The attack threat $T_{i,cI}^{j}$ posed by the movement $P_{i,cI}^{j}$ is equivalent to that of the distributed offensive threat-I and is simulated using Eq. (9).

#### Distributed offensive threat-II

To secure a victory in a chess game, the utilization of dispersed offensive threats cannot be confined to a single mode; it necessitates appropriate modifications to yield unanticipated outcomes. While the global optimal solution can offer comprehensive guidance for each movement step, the occasional implementation of a retreat strategy can also pave the way for the ultimate triumph. In this regard, the influence of the global optimal solution in the decentralized offensive threat I on the new position is eliminated, and the new position $P_{i,dII}^{j}$ is determined solely by the positions of a random individual and the currently updated individual. For this simulation, Eq. (12) is employed.

12 $$\begin{aligned} P_{i,dII}^{j}=x_{i}^{j}+0.7\times (x_{i}^{j}-x_{r}^{j}) \end{aligned}$$

The attack threat $T_{i,dII}^{j}$ caused by the movement of $P_{i,dII}^{j}$ is the same as that of the distributed offensive threat-I and is simulated using Eq. (9).

#### Concentrated offensive threat-II

Analogous to distributed offensive threats, concentrated offensive threats also necessitate diversification. In this context, we introduce Concentrated Offensive Threat II. In contrast to Concentrated Offensive Threat I, the influence of the global optimal solution is also eliminated in this design. Upon removal, the new location $P_{i,cII}^{j}$ becomes equivalent to the location of the currently updated individual, as depicted in Eq. (13).

13 $$\begin{aligned} P_{i,cII}^{j}=x_{i}^{j} \end{aligned}$$

The attack threat caused by the movement of $P_{i,cII}^{j}$ was simulated using Eq. (14).

14 $$\begin{aligned} T_{i,p}^{j}=r\times (x_{gb}^{j}-x_{i}^{j}) \end{aligned}$$

where *r* is a uniformly distributed random number within the interval [0,1].

#### Total offensive strategy

**Fig. 1:**
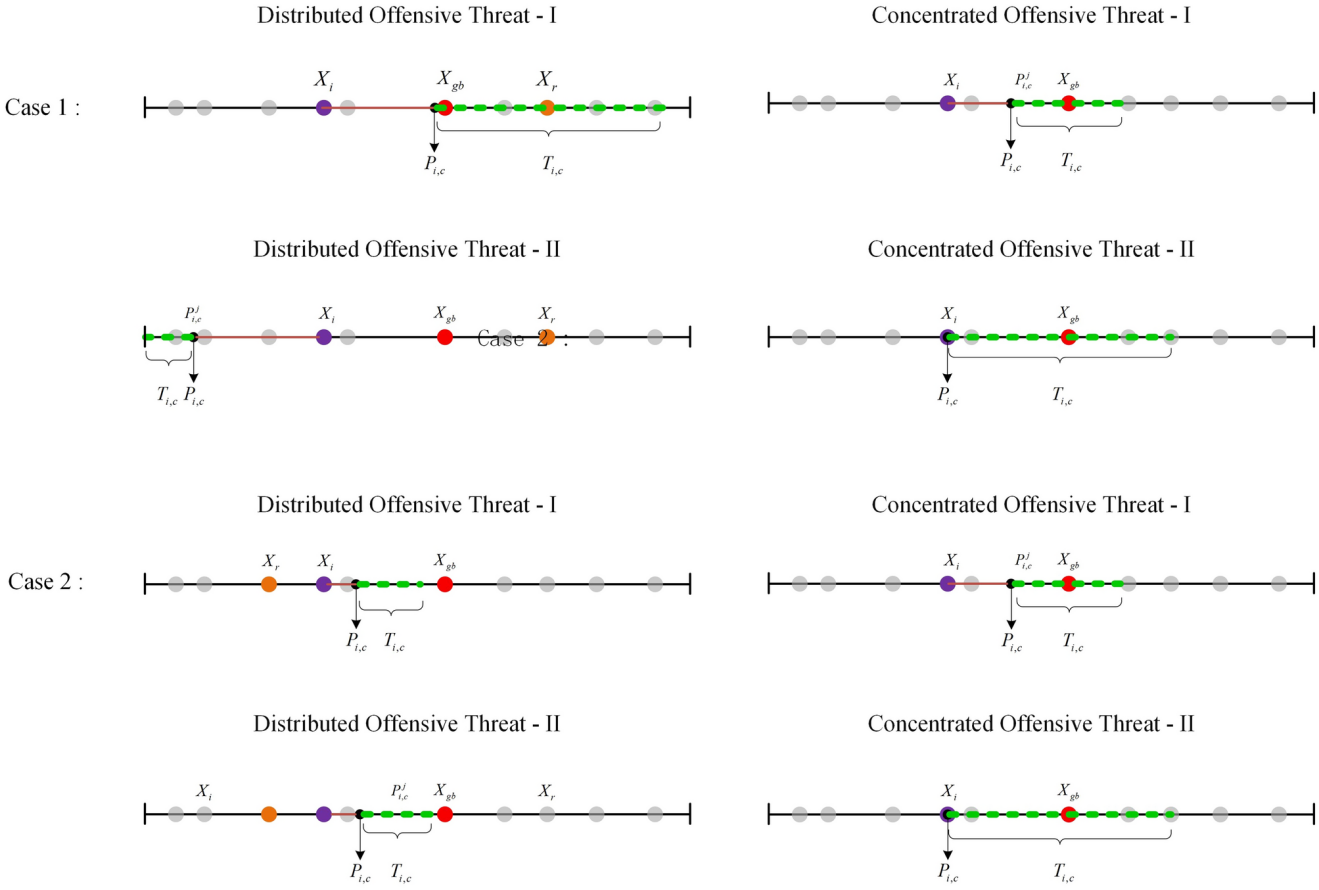
Comparison of threat differences caused by four offensive strategies in two cases.

Figure 1 illustrates the variations in mobile threats engendered by four distinct attack strategies, given the same current individual position and the same random individual. The bold line in the figure demarcates the potential reach of individuals within the population under a specific mobile threat. Evaluating from the standpoint of the diversity of the area that individuals can access, the Distributed Offensive Threat-I exhibits the greatest extent, followed by the Distributed Offensive Threat-II. The Concentrated Offensive Threat-I and the Concentrated Offensive Threat-II are comparable in this regard. In terms of the range of areas that individuals can access, the Distributed Offensive Threat-I surpasses the Distributed Offensive Threat-II, with the Concentrated Offensive Threat-II and the Concentrated Offensive Threat-I trailing behind. Furthermore, while the four attack strategies primarily cater to the global search, they also contribute to the local search. When compared with the Distributed Offensive Threats, the Concentrated Offensive Threats demonstrate a greater inclination towards local search.

Given the distinct effects of the four attack strategies, a probability selection method can be employed during the search process to execute a crossover operation, with a roulette mechanism determining the attack method. The primary objective of the attack is to facilitate global search, and modes that can diversify large-scale search are prioritized for execution. Consequently, Distributed Offensive Threat-I emerges as the preferred method. To circumvent the risk of the algorithm converging to local minima due to a singular search behavior, both Distributed Offensive Threats and Concentrated Offensive Threats adopt a crossover selection strategy. The overarching offensive strategy is encapsulated in Eq. (15).

15 $$\begin{aligned} \begin{aligned} x_{new}^{j}= {\left\{ \begin{array}{ll} P_{i,dI}^{j}+T_{i,dI}^{j} & \quad if\, \, {{r}_{1}}<{{p}_{1}} \\ P_{i,cI}^{j}+T_{i,cI}^{j} & \quad else\, \, if\, {{r}_{2}}<{{p}_{1}}\\ P_{i,dII}^{j}+T_{i,dII}^{j} & \quad else\, if\, {{r}_{3}}<{{p}_{1}}\\ P_{i,cII}^{j}+T_{i,cII}^{j} & \quad else \end{array}\right. } \end{aligned} \end{aligned}$$

where ${{p}_{1}}$ denotes the offensive selection probability, possessing a value of 0.7 in this paper.

Upon each individual securing a new position within the solution space via the offensive strategy, the method of update is ascertained based on the fitness value. This process is encapsulated in the subsequent equation.

16 $$\begin{aligned} \begin{aligned} {{X}_{i}}= {\left\{ \begin{array}{ll} {{X}_{new}} & \quad if\, f({{X}_{new}})<f({{X}_{i}}) \\ {{X}_{i}} & \quad otherwise \end{array}\right. } \end{aligned} \end{aligned}$$

### Defensive strategy

**Fig. 2:**
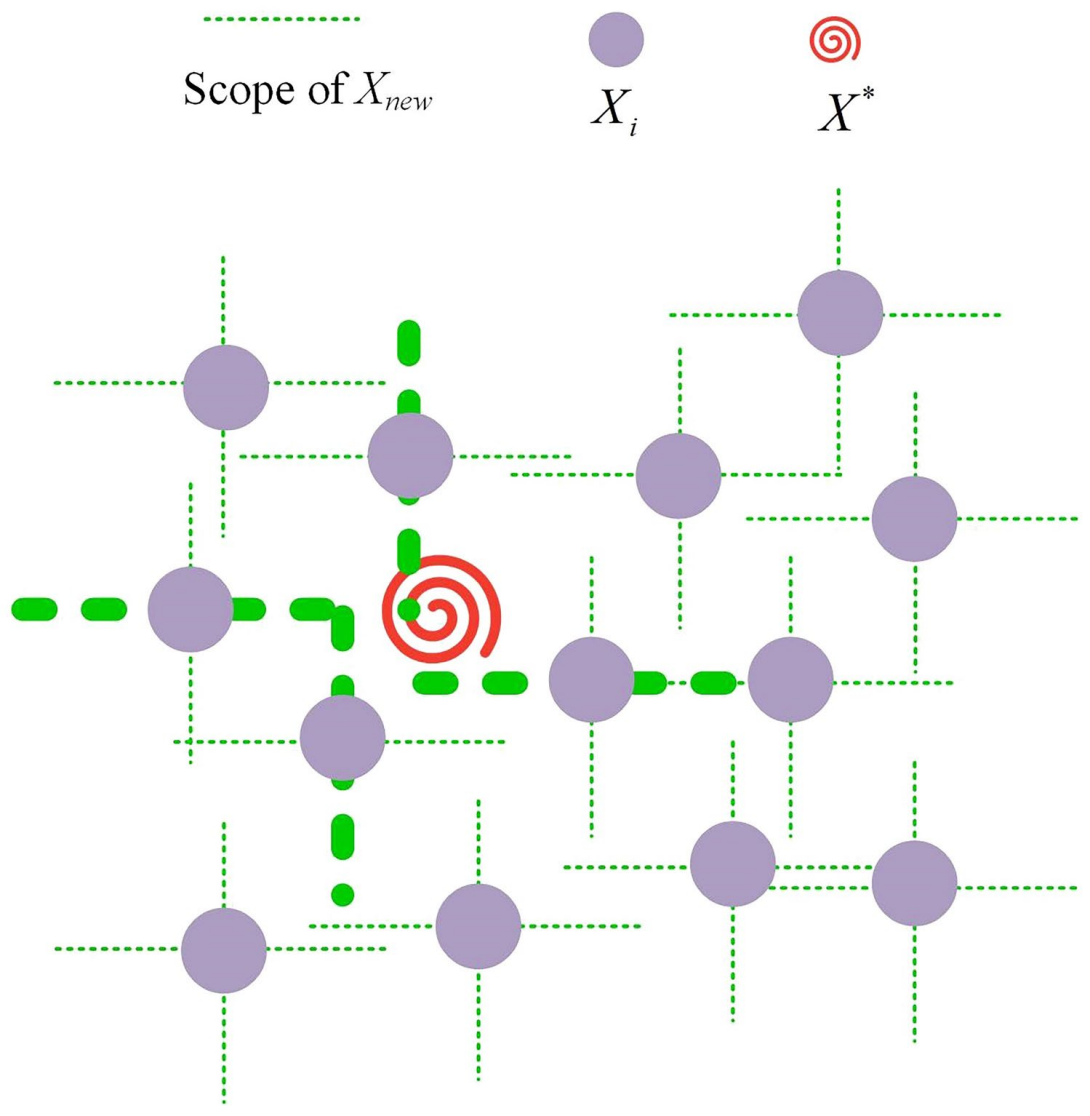
Schematic diagram of movement effect of individuals in some dimensions under defensive strategy.

Defense involves identifying opportune moments to launch counterattacks while simultaneously defending against and resisting the adversary’s offensive maneuvers. Given that an individual will traverse all dimensions during an attack, the defensive strategy predominantly remains stationary across most dimensions, with alterations occurring in a select few dimensions during an assault or counterattack. As depicted in Fig. 2, each individual within the population in the problem solution space possesses the potential to transition to the optimal solution position. However, this necessitates movement along a specific dimension, as opposed to all dimensions. This scenario is particularly applicable when the population has evolved to a certain stage and is in proximity to the optimal solution, leveraging the current positional advantage of the population.

The aforementioned search behavior is referred to as Assault Defense, and the symbol $X_{new}^{d}=(x_{new}^{1},x_{new}^{2},\ldots ,x_{new}^{D})$ is employed to denote the results of the defense. The specific alterations in the value of dimension *j* are encapsulated in Eq. (17). This modification of select dimensions underscores that this search behavior is characteristic of a local search mode.

17 $$\begin{aligned} x_{new}^{i}=\left\{ \begin{array}{ll} x_{i}^{j}+2r\times (x_{r}^{j}-x_{i}^{j}) & \quad if\, {{r}_{4}}<{{p}_{2}} \\ x_{i}^{j} & \quad otherwise \\ \end{array} \right. \end{aligned}$$

where ${{r}_{4}}$ denotes a uniformly distributed random number within interval [0,1]. ${{p}_{2}}$ signifies the probability of remaining in its original state or moving, with a fixed value of 0.3.

To circumvent the risk of the algorithm converging to local minima, it is imperative for each strategy to maintain the diversity of population movement. The attack strategy fosters this diversity through four distinct modes of movement. In the context of the defense strategy, the singular partial dimension movement coupled with the preferred update position strategy may hinder the algorithm’s ability to escape local minima. Given that the defense strategy is primarily geared towards local search, a probability mechanism is employed to establish a reset defense behavior by randomly initializing values across certain dimensions, as depicted in Eq. (18). It is a requisite that individuals executing the reset defensive behavior transition to a new position, thereby enhancing the diversity within the population.

18 $$\begin{aligned} x_{new}^{i}=\left\{ \begin{array}{ll} LB_{ }^{j}+r\times (UB_{ }^{j}-LB_{ }^{j}) & \quad if\, {{r}_{5}}<{{p}_{3}} \\ x_{i}^{j} & \quad otherwise \\ \end{array} \right. \end{aligned}$$

where the parameter ${{p}_{3}}$ assumes a value of 0.1, denoting the probability associated with the resetting of a specific dimension value, commonly referred to as the reset defense dimension selection probability. Additionally, variable ${{r}_{5}}$ is a random number uniformly distributed within the range of [0,1].

The Assault Defense and Reset Defense collectively constitute the comprehensive defensive behavior. The execution of either strategy is determined probabilistically, with the probability ${{p}_{4}}$ denoting the likelihood of executing the Reset Defense. In comparison to the Assault Defense, the Reset Defense exhibits greater randomness in its movement objectives, lacking a definitive destination. This approach is conducive to enhancing diversity within the population during defense, albeit it is not optimal for deep local search. The probability of identifying the problem’s optimal solution is relatively low. Given this consideration, ${{p}_{4}}$ should not assume a large value. In the context of this study, ${{p}_{4}}$ is set to 0.01.

### Flowchart

The ODO algorithm employs the initialization technique commonly utilized by the majority of metaheuristic algorithms during the initial phase of population initialization, as demonstrated in Eq. (19).

19 $$\begin{aligned} X=LB+r\cdot (UB-LB) \end{aligned}$$

The ODO algorithm emulates the process of playing chess, mirroring the strategic maneuvers of a chess player. The dynamics of a chess game are in constant flux, rendering the determination of specific steps for attack and defense ambiguous. Consequently, during the iterative process, the execution of each strategy is governed by a probabilistic mechanism. Despite the random selection of the specific strategy to be implemented in each iteration, the overarching strategy of chess gameplay can be encapsulated as “initial dispersion, subsequent concentration, dispersion threatening concentration, and ultimate victory through concentration.” In accordance with this core principle, the choice between attack and defense is contingent upon the adaptive factor $\omega$, as delineated in Eq. (20).

20 $$\begin{aligned}&{{X}_{new}}=\left\{ \begin{array}{ll} X_{new}^{a} & \quad if\, {{r}_{6}}<\omega \\ X_{new}^{d} & \quad otherwise \\ \end{array} \right. \end{aligned}$$

21 $$\begin{aligned}&\omega ={{\omega }_{\max }}-t\cdot ({{\omega }_{\max }}-{{\omega }_{\min }})/{{T}_{\max }} \end{aligned}$$

where ${{\omega }_{\max }}$ and ${{\omega }_{\min }}$ represent the maximum and minimum values of the adaptive factor, which are 0.9 and 0.2, respectively, *t* is the current number of iterations, and ${{T}_{\max }}$ is the total number of iterations. ${{r}_{6}}$ is a uniformly distributed random number in the interval [0,1], ${X_{new}^{a}}$ is the result of the Offensive, ${X_{new}^{d}}$ is the result of the defense.

The update rule for the global optimal solution resembles the update method employed for the position of the individual, as depicted in the subsequent equation.

22 $$\begin{aligned} \begin{aligned} {{X}_{gb}}= {\left\{ \begin{array}{ll} {{X}_{new}} & if\, f({{X}_{new}})<f({{X}_{gb}}) \\ {{X}_{gb}} & otherwise \end{array}\right. } \end{aligned} \end{aligned}$$

**Fig. 3:**
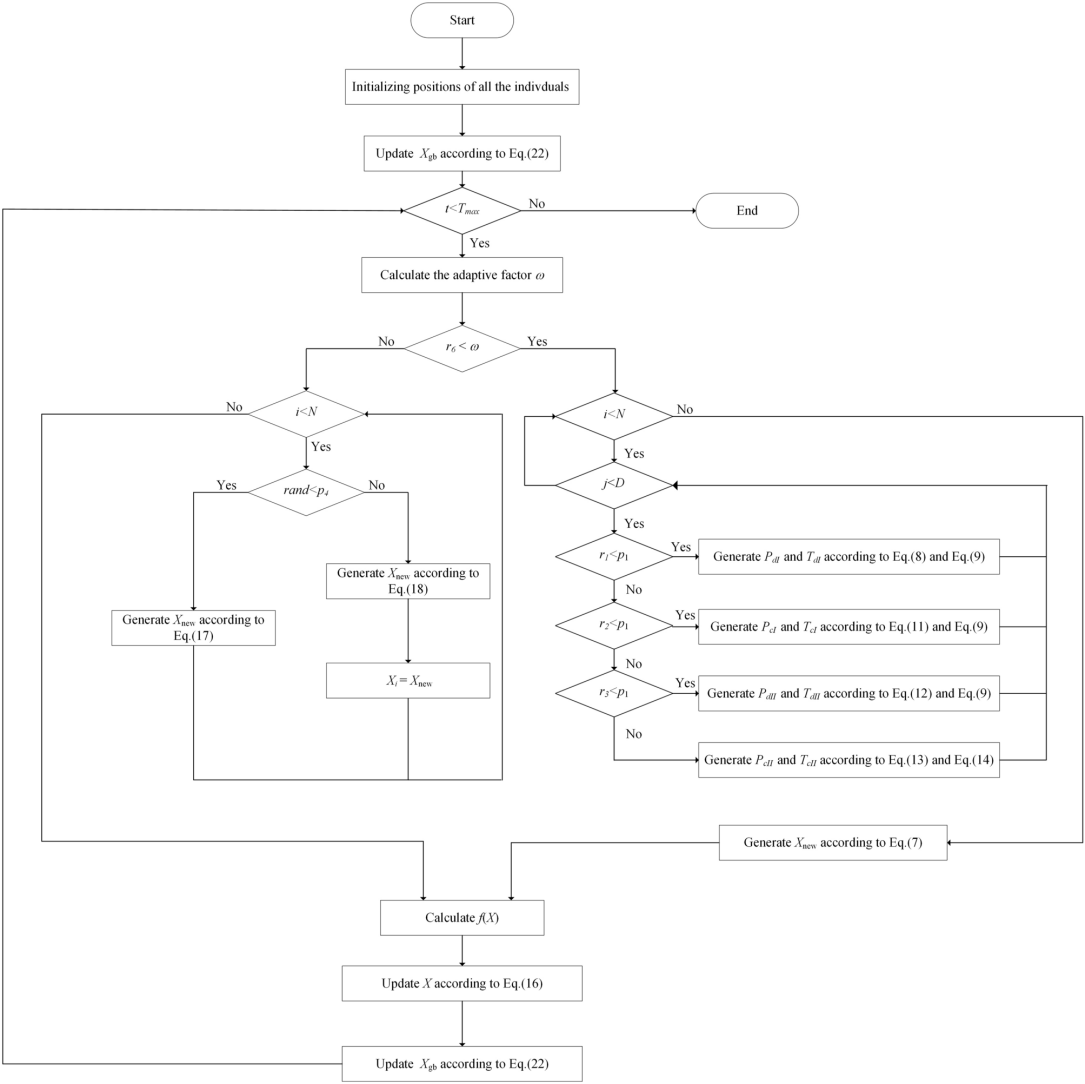
Flowchart of ODO.

The aforementioned processes encompass the entire ODO algorithm flowchart, as depicted in Fig. 3.

### Computational complexity

The computational complexity of the proposed ODO algorithm is contingent upon three key factors: the initialization process, the evaluation of the fitness function, and the solution update. The complexity of the initialization process can be expressed as a function of the number of individuals, denoted by ${\mathrm O}(N)$. The complexity of the fitness function evaluation is problem-dependent and will not be elaborated upon further in this discussion. Finally, the complexity of updating the solution can be expressed as a function of the number of iterations, denoted by ${\mathrm O}(N\times {{T}_{\max }})$ . Consequently, the overall computational complexity of the proposed ODO algorithm can be determined by ${\mathrm O}(N\times ({{T}_{\max }}+1))$. In the subsequent sections, a range of benchmark functions and real-world optimization problems are employed to validate and substantiate the efficacy of ODO.

### Exploration ability of ODO

In the context of the Offensive Defensive Optimization (ODO) algorithm, the exploration component is predominantly facilitated by the offensive strategy. Influenced by the adaptive parameter $\omega$, the probability of execution is high at the onset of the iteration, thereby enabling rapid convergence. Towards the end of the iteration, the probability of execution diminishes, serving to augment the diversity of search behavior for exploitation. The parameter $\omega$ exhibits a linear variation with the number of iterations, striking a balance between exploration and exploitation, without an overemphasis on either global or local searches.

The parameter ${{p}_{1}}$ primarily governs the specific search behavior enacted by the Offensive Defensive Optimization during the exploration phase, determining the sequence of attack behaviors. It is crucial that the value of ${{p}_{1}}$ is not excessively small, ensuring that the execution instances of Distributed Offensive Threat-I surpass the combined total of the other three. This facilitates the global search in identifying the potential optimal solution or a solution proximate to the optimal solution across a broader space, thereby offering a more effective search space for local search. The remaining three attack modes contribute to the diversity of individual movement during exploration, enabling each individual to enhance the diversity of their mobile destination through combined movement. The cross-selection mode is also configured to augment the diversity of movement destinations.

### Exploitation ability of ODO

In the context of the Offensive Defensive Optimization algorithm, the exploitation component is predominantly facilitated by the defensive strategy, signifying local search behavior. The local search is contingent upon the location of the core search. The Assault Defense strategy is designed to conduct searches in the vicinity of each chess piece within the group. Consequently, the parameter ${{p}_{2}}$ is employed to regulate whether an individual remains stationary or necessitates movement along a specific dimension. It is crucial that the value of ${{p}_{2}}$ is not excessively large, as this would bias the movement outcome towards exploration. The advantage of conducting searches near each chess piece is that it enables the algorithm to detect subtle trend changes within a limited range. For a solution proximate to a peak in the solution space, it can identify a superior solution. For a solution situated between multiple dense peaks in the solution space, it can assist in escaping local minima and identifying a superior solution. In contrast to the Assault Defense, the Reset Defense concentrates on the sporadic random movement along a specific dimension for local search. Therefore, the value of ${{p}_{3}}$ should not be excessively large and should be less than ${{p}_{2}}$. This operation increases the probability of the algorithm escaping local minima towards the end of the iteration. Moreover, given the high degree of randomness associated with the Reset Defense method, excessive execution could disrupt the movement trend of the population, necessitating a very small value for ${{p}_{3}}$.

## Calculation and analysis

### Calculation setup

This section presents a comprehensive analysis of the ODO algorithm. The validity of the algorithm was established through its application to a benchmark test set comprising 41 real-parameter single-objective optimizations sourced from the widely recognized CEC2017 and CEC2022 test sets^27^. These test sets are commonly employed to evaluate the efficacy of optimization algorithms, encompassing 29 benchmarks from CEC2017 and 12 benchmarks from CEC2022. To facilitate the presentation of the outcomes, the benchmark function of the CEC2017 test set is designated as F1–F29, where F1 and F2 represent unimodal functions, F3–F9 signifying multimodal functions, F10–F19 denoting mixed functions, and F20–F29 representing composite functions. Similarly, the CEC2022 test set function was denoted as F30–F41, where F30 corresponds to a unimodal function, F31–F34 represents multimodal functions, F35–F37 signifies mixed functions, and F38–F41 denotes composite and multimodal functions. Unimodal functions are distinguished by the absence of local minima with only a global minimum present, thereby serving as a means of assessing the convergence capability of the algorithm. Conversely, multimodal functions possess local extremum points, which are employed to evaluate the capacity of the algorithm to escape from local optima. A mixed function, on the other hand, encompasses three or more benchmark functions following rotation or displacement, with each sub function being assigned a specific weight. Composite functions possess the attribute of being comprised of a minimum of three mixed or benchmark functions that have undergone rotation and shifting. In addition, each subfunction within the composite function is accompanied by an extra bias value and weight. Consequently, these composite functions exacerbate the complexity of the algorithmic optimization.

The experiments were carried out using MATLAB R2020a on a personal computer equipped with an Intel (R) Core (TM) i5-7200U CPU @ 2.50GHz 2.71 GHz and a Microsoft Windows 10 Enterprise 64-bit operating system. For the algorithm validation, 30 independent runs were conducted to account for the randomness of the algorithm. The conclusions were derived from the results of these runs, including the best solution (BEST), mean (AVG), and standard deviation (STD) obtained for each iteration. This study examined the varying dimensions of the two test sets, namely the CEC2017 and CEC2022 test sets. Specifically, the dimensions considered were 10, 30, 50, and 100 for the CEC2017 test set and 10 and 20 for the CEC2022 test set. For a fair comparison, all algorithms were tested with identical settings: a population size of 30 and 5000 iterations, resulting in 150,000 total function evaluations.

To verify the superiority of the algorithm, some classic algorithms and recently published algorithms were compared, namely Particle Swarm Optimization (PSO)^5^, teaching learning-based optimization (TLBO)^33^, Fox Optimizer (FOX)^47^ , Snake Optimizer (SO)^48^, Sea-horse Optimizer (SHO)^49^, IbI logic algorithm (ILA)^43^, Group Teaching Optimization Algorithm with Information Sharing (ISGTOA)^38^, and enhanced snake optimizer (ESO)^50^ . The parameter settings of the algorithms are listed in Table 1. It is worth noting that the codes for these selected comparison methods have been published online by the authors of the article.

**Table 1:** The default parameter settings of the rival optimizers.

Algorithms	The default parameter settings
PSO^5^	$\omega$: linear reduction from 0.9 to 0.2; (${{C}_{1}}$,${{C}_{2}}$): (2,2); ${{V}_{max}}$: 6; ${{V}_{min}}$: −6
TLBO^33^	${{T}_{F}}$: 1 or 2
FOX^47^	${{c}_{1}}$: 0.18 ; ${{c}_{2}}$: 0.82
SO^48^	${{T}_{1}}$: 0.25; ${{T}_{2}}$: 0.6; ${{C}_{1}}$: 0.5; ${{C}_{2}}$: 0.05; ${{C}_{3}}$: 2
SHO^49^	${{r}_{1}}$: 0; ${{r}_{2}}$: 0.1
ILA^43^	${{n}_{m}}$: 5; ${{p}_{s1}}$: 0.33; ${{p}_{s2}}$: 0.33; ${{B}_{min}}$: 0.4; ${{B}_{max}}$: 0.6; ${{n}_{rep}}$: 10; ${{t}_{clus}}$: 100
ISGTOA^38^	NA
ESO^50^	${{T}_{1}}$: 0.25; ${{T}_{2}}$: 0.6; ${{C}_{1}}$: 0.5; ${{C}_{2}}$: 0.05; ${{C}_{3}}$: 2

### Accuracy analysis

To ascertain the comparative excellence of the proposed ODO algorithm on the CEC2017 test set, Table 2 presents the optimal solution, average solution, and standard deviation achieved by each competing algorithm in 30 separate runs for each benchmark function on the 30 dimensions. Subsequently, Friedman tests were conducted using the optimal solution obtained from the 30 independent runs to obtain rank-ranking outcomes. Based on the sorting results presented in Table 2, it is evident that the ODO algorithm demonstrates superior performance across the majority of the test functions, securing the top position. This outcome underscores the strong competitiveness of the ODO algorithm in the context of the 30 dimensions. Additionally, the PSO, TLBO, SO, ISGTOA, FOX, and ILA also achieved notable success on several specific test functions, highlighting the distinct optimization capabilities possessed by each exceptional algorithm.

**Table 2:** Optimization results for CEC2017 test suit with dimension of 30. Significant values are in bold.

Function	Measure	ODO	PSO	TLBO	FOX	SO	SHO	ILA	ISGTOA	ESO
F1	Best	**1.00E+02**	1.09E+02	1.10E+02	3.20E+02	1.24E+02	1.62E+10	8.89E+07	1.07E+02	1.73E+05
	Mean	**1.00E+02**	6.85E+07	3.36E+03	1.68E+03	7.17E+03	2.42E+10	1.66E+08	1.30E+03	1.37E+06
	Std	**6.36E**−**03**	2.61E+08	4.06E+03	8.65E+02	6.20E+03	4.20E+09	6.38E+07	1.41E+03	1.51E+06
	Rank	**1**	4	5	3	6	9	8	2	7
F2	Best	2.00E+02	2.00E+02	2.02E+02	2.00E+02	1.41E+03	5.06E+04	7.91E+03	**2.00E+02**	7.77E+03
	Mean	2.09E+02	2.00E+02	2.64E+02	**2.00E+02**	1.06E+04	6.48E+04	1.44E+04	2.00E+02	1.52E+04
	Std	1.65E+01	2.37E−02	8.70E+01	**6.17E**−**03**	5.58E+03	7.61E+03	4.17E+03	2.51E−01	4.60E+03
	Rank	4	2	5	3	6	9	7	**1**	8
F3	Best	**3.00E+02**	3.04E+02	3.00E+02	3.04E+02	3.04E+02	1.68E+03	4.21E+02	3.00E+02	3.07E+02
	Mean	**3.26E+02**	3.93E+02	3.88E+02	3.70E+02	3.83E+02	4.11E+03	4.54E+02	3.68E+02	4.05E+02
	Std	3.57E+01	3.39E+01	2.52E+01	2.84E+01	2.25E+01	1.72E+03	**2.09E+01**	2.59E+01	2.68E+01
	Rank	**1**	5	6	3	4	9	8	2	7
F4	Best	**4.45E+02**	5.10E+02	4.85E+02	6.91E+02	4.53E+02	6.71E+02	4.91E+02	4.65E+02	4.83E+02
	Mean	**4.81E+02**	5.89E+02	5.06E+02	7.06E+02	5.00E+02	7.24E+02	5.81E+02	5.12E+02	5.33E+02
	Std	1.99E+01	3.39E+01	**1.67E+01**	1.84E+01	1.73E+01	2.40E+01	4.75E+01	3.10E+01	2.30E+01
	Rank	**1**	7	3	8	2	9	6	4	5
F5	Best	**5.00E+02**	5.19E+02	5.04E+02	5.60E+02	5.01E+02	5.57E+02	5.13E+02	5.03E+02	5.02E+02
	Mean	**5.00E+02**	5.41E+02	5.17E+02	5.73E+02	5.07E+02	5.72E+02	5.33E+02	5.13E+02	5.19E+02
	Std	**0.00E+00**	9.48E+00	7.10E+00	8.92E+00	4.79E+00	7.00E+00	1.02E+01	6.16E+00	9.21E+00
	Rank	**1**	7	4	8	2	9	6	3	5
F6	Best	**6.62E+02**	6.93E+02	7.08E+02	1.18E+03	6.66E+02	1.03E+03	7.65E+02	7.11E+02	7.06E+02
	Mean	**7.17E+02**	7.37E+02	7.99E+02	1.22E+03	7.26E+02	1.13E+03	8.25E+02	7.70E+02	7.64E+02
	Std	2.59E+01	3.66E+01	5.52E+01	**1.50E+01**	2.97E+01	5.21E+01	2.65E+01	3.99E+01	4.76E+01
	Rank	**1**	3	6	9	2	8	7	5	4
F7	Best	**7.44E+02**	7.87E+02	7.45E+02	8.81E+02	7.61E+02	9.10E+02	8.10E+02	7.62E+02	7.60E+02
	Mean	**7.77E+02**	8.30E+02	7.86E+02	8.98E+02	7.98E+02	9.53E+02	8.64E+02	8.04E+02	8.05E+02
	Std	2.14E+01	2.55E+01	1.94E+01	**1.68E+01**	2.12E+01	2.26E+01	2.45E+01	2.03E+01	2.01E+01
	Rank	**1**	6	2	8	3	9	7	5	4
F8	Best	**8.02E+02**	1.55E+03	9.39E+02	5.24E+03	8.81E+02	5.80E+03	1.25E+03	9.50E+02	8.07E+02
	Mean	**8.57E+02**	3.66E+03	1.55E+03	5.33E+03	1.66E+03	7.78E+03	2.99E+03	1.54E+03	2.41E+03
	Std	**4.22E+01**	9.21E+02	5.06E+02	5.58E+01	6.50E+02	9.45E+02	1.40E+03	3.72E+02	1.56E+03
	Rank	**1**	7	3	8	4	9	6	2	5
F9	Best	2.32E+03	3.45E+03	3.46E+03	4.09E+03	2.59E+03	5.93E+03	3.36E+03	3.80E+03	**2.21E+03**
	Mean	3.87E+03	4.43E+03	7.06E+03	5.52E+03	**3.46E+03**	6.94E+03	4.58E+03	5.57E+03	3.66E+03
	Std	1.59E+03	6.27E+02	1.25E+03	6.38E+02	**4.23E+02**	5.71E+02	5.17E+02	1.00E+03	1.12E+03
	Rank	3	4	9	7	**1**	8	5	6	2
F10	Best	**1.03E+03**	1.05E+03	1.06E+03	1.07E+03	1.06E+03	2.32E+03	1.19E+03	1.06E+03	1.08E+03
	Mean	**1.09E+03**	1.11E+03	1.13E+03	1.19E+03	1.15E+03	4.38E+03	1.27E+03	1.12E+03	1.14E+03
	Std	4.03E+01	**3.28E+01**	3.53E+01	5.79E+01	5.76E+01	1.65E+03	4.26E+01	4.45E+01	3.80E+01
	Rank	**1**	2	4	7	5	9	8	3	6
F11	Best	6.02E+03	1.20E+04	**4.95E+03**	1.35E+05	2.23E+04	7.19E+08	5.56E+06	9.34E+03	1.83E+05
	Mean	**2.30E+04**	5.80E+05	5.84E+04	1.55E+06	4.61E+05	2.75E+09	3.44E+07	2.73E+04	8.79E+06
	Std	**9.97E+03**	2.38E+06	6.83E+04	1.45E+06	5.51E+05	1.31E+09	1.95E+07	1.12E+04	7.20E+06
	Rank	**1**	4	3	6	5	9	8	2	7
F12	Best	**1.30E+03**	1.54E+03	2.23E+03	2.05E+04	3.15E+03	8.14E+07	1.19E+05	2.01E+03	2.56E+04
	Mean	**1.50E+04**	1.60E+04	2.32E+04	1.09E+05	2.63E+04	1.47E+09	5.62E+05	1.77E+04	3.72E+05
	Std	**1.43E+04**	1.60E+04	1.88E+04	7.97E+04	2.12E+04	1.57E+09	6.68E+05	1.64E+04	3.71E+05
	Rank	2	**1**	4	6	5	9	8	3	7
F13	Best	**1.34E+03**	1.49E+03	1.64E+03	1.72E+03	1.61E+03	1.05E+05	3.18E+03	1.39E+03	1.77E+03
	Mean	1.58E+03	8.97E+03	6.53E+03	5.98E+03	8.17E+03	5.72E+05	2.96E+04	**1.53E+03**	5.61E+03
	Std	2.49E+02	5.83E+03	4.61E+03	2.70E+03	5.53E+03	2.30E+05	4.77E+04	**8.27E+01**	3.37E+03
	Rank	**1**	7	4	5	6	9	8	2	3
F14	Best	**1.41E+03**	1.60E+03	1.71E+03	1.03E+04	1.60E+03	4.06E+05	2.03E+04	1.56E+03	2.44E+03
	Mean	**1.50E+03**	1.09E+04	4.90E+03	2.96E+04	1.15E+04	5.78E+06	5.27E+04	7.05E+03	3.39E+04
	Std	**2.13E+02**	1.18E+04	4.46E+03	3.58E+04	1.13E+04	1.67E+07	2.85E+04	6.29E+03	2.90E+04
	Rank	**1**	4	2	6	5	9	8	3	7
F15	Best	1.86E+03	2.23E+03	1.79E+03	2.25E+03	1.90E+03	2.71E+03	2.01E+03	**1.67E+03**	1.97E+03
	Mean	2.28E+03	2.65E+03	**2.23E+03**	3.38E+03	2.34E+03	3.46E+03	2.58E+03	2.38E+03	2.55E+03
	Std	**2.36E+02**	2.95E+02	2.38E+02	4.71E+02	2.62E+02	2.89E+02	2.43E+02	3.05E+02	2.44E+02
	Rank	2	7	1	8	3	9	6	4	5
F16	Best	1.65E+03	1.67E+03	**1.65E+03**	1.83E+03	1.68E+03	1.99E+03	1.69E+03	1.72E+03	1.73E+03
	Mean	1.89E+03	2.29E+03	**1.88E+03**	2.62E+03	2.04E+03	2.37E+03	1.96E+03	2.01E+03	1.98E+03
	Std	1.63E+02	2.89E+02	1.49E+02	3.78E+02	1.80E+02	2.17E+02	1.46E+02	1.81E+02	**1.37E+02**
	Rank	2	7	**1**	9	6	8	3	5	4
F17	Best	**2.71E+03**	4.17E+04	3.80E+04	4.34E+04	4.44E+04	3.08E+05	5.26E+04	6.09E+03	3.22E+04
	Mean	**1.58E+04**	1.33E+05	2.73E+05	2.00E+05	1.90E+05	2.17E+06	3.41E+05	4.10E+04	1.26E+05
	Std	**1.46E+04**	8.51E+04	1.86E+05	1.23E+05	1.23E+05	2.53E+06	3.41E+05	2.71E+04	5.44E+04
	Rank	**1**	3	8	5	6	9	7	2	4
F18	Best	**1.81E+03**	1.92E+03	1.89E+03	1.20E+05	2.01E+03	1.38E+06	4.35E+04	1.89E+03	3.32E+03
	Mean	**1.89E+03**	8.72E+03	6.77E+03	2.03E+05	1.43E+04	1.66E+07	7.49E+05	3.26E+03	5.35E+05
	Std	**2.25E+02**	7.71E+03	5.87E+03	1.18E+05	1.29E+04	3.54E+07	7.06E+05	2.48E+03	9.59E+05
	Rank	**1**	3	4	7	5	9	8	2	6
F19	Best	**1.92E+03**	2.18E+03	2.03E+03	2.47E+03	2.00E+03	2.31E+03	2.07E+03	1.99E+03	2.02E+03
	Mean	**2.11E+03**	2.53E+03	2.20E+03	2.92E+03	2.36E+03	2.55E+03	2.30E+03	2.33E+03	2.27E+03
	Std	1.15E+02	2.15E+02	1.13E+02	2.69E+02	1.86E+02	1.70E+02	**9.49E+01**	1.88E+02	1.38E+02
	Rank	**1**	7	2	9	6	8	5	4	3
F20	Best	2.24E+03	2.29E+03	2.25E+03	2.47E+03	2.24E+03	2.39E+03	**2.15E+03**	2.26E+03	2.27E+03
	Mean	**2.28E+03**	2.38E+03	2.29E+03	2.59E+03	2.30E+03	2.48E+03	2.31E+03	2.29E+03	2.31E+03
	Std	**1.78E+01**	4.38E+01	2.57E+01	5.40E+01	1.95E+01	2.67E+01	7.67E+01	2.71E+01	2.43E+01
	Rank	**1**	7	2	9	4	8	6	3	5
F21	Best	**2.20E+03**	**2.20E+03**	**2.20E+03**	5.40E+03	2.20E+03	4.06E+03	2.25E+03	**2.20E+03**	2.20E+03
	Mean	2.34E+03	5.25E+03	2.22E+03	7.40E+03	3.81E+03	6.59E+03	2.28E+03	3.65E+03	**2.21E+03**
	Std	5.42E+02	1.94E+03	8.01E+01	1.05E+03	1.46E+03	1.81E+03	2.01E+01	2.01E+03	**3.75E+00**
	Rank	**1**	7	2	9	5	8	6	4	3
F22	Best	**2.59E+03**	2.85E+03	2.62E+03	3.01E+03	2.62E+03	2.91E+03	2.66E+03	2.61E+03	2.63E+03
	Mean	**2.64E+03**	3.07E+03	2.69E+03	3.42E+03	2.69E+03	3.00E+03	2.72E+03	2.67E+03	2.70E+03
	Std	**2.98E+01**	1.28E+02	3.73E+01	1.87E+02	4.06E+01	4.75E+01	3.39E+01	3.90E+01	4.73E+01
	Rank	**1**	8	3	9	4	7	6	2	5
F23	Best	2.78E+03	2.95E+03	2.77E+03	3.24E+03	2.79E+03	3.11E+03	2.81E+03	**2.76E+03**	2.78E+03
	Mean	2.85E+03	3.09E+03	2.84E+03	3.55E+03	2.84E+03	3.23E+03	2.87E+03	**2.83E+03**	2.83E+03
	Std	4.41E+01	7.60E+01	3.74E+01	1.14E+02	**2.57E+01**	6.20E+01	2.97E+01	3.95E+01	2.85E+01
	Rank	5	7	3	9	4	8	6	**1**	2
F24	Best	2.78E+03	2.78E+03	2.78E+03	**2.78E+03**	2.78E+03	3.12E+03	2.81E+03	2.78E+03	2.79E+03
	Mean	2.80E+03	2.79E+03	2.81E+03	2.79E+03	**2.79E+03**	3.40E+03	2.86E+03	2.81E+03	2.81E+03
	Std	1.77E+01	1.40E+01	2.24E+01	9.94E+00	**1.38E+00**	2.05E+02	2.59E+01	2.05E+01	2.08E+01
	Rank	4	3	6	**1**	2	9	8	5	7
F25	Best	**2.70E+03**	**2.70E+03**	**2.70E+03**	3.72E+03	4.44E+03	5.50E+03	2.88E+03	4.20E+03	4.63E+03
	Mean	4.49E+03	5.67E+03	5.25E+03	8.04E+03	5.55E+03	7.81E+03	**3.26E+03**	5.18E+03	6.18E+03
	Std	1.32E+03	1.97E+03	1.20E+03	1.41E+03	5.45E+02	8.58E+02	**5.35E+02**	5.98E+02	8.67E+02
	Rank	2	6	4	8	5	9	**1**	3	7
F26	Best	**3.11E+03**	3.14E+03	3.13E+03	3.31E+03	3.14E+03	3.37E+03	3.13E+03	3.11E+03	3.12E+03
	Mean	**3.15E+03**	3.31E+03	3.17E+03	4.05E+03	3.19E+03	3.53E+03	3.17E+03	3.16E+03	3.17E+03
	Std	**2.02E+01**	1.40E+02	3.08E+01	4.71E+02	2.77E+01	1.27E+02	2.35E+01	2.89E+01	2.97E+01
	Rank	**1**	7	4	9	6	8	5	2	3
F27	Best	**3.00E+03**	**3.00E+03**	3.00E+03	3.00E+03	3.09E+03	3.74E+03	3.20E+03	**3.00E+03**	3.10E+03
	Mean	3.03E+03	3.12E+03	3.11E+03	**3.01E+03**	3.12E+03	4.39E+03	3.24E+03	3.10E+03	3.15E+03
	Std	5.59E+01	3.32E+01	3.28E+01	2.97E+01	**1.50E+01**	3.78E+02	2.32E+01	5.01E+01	2.08E+01
	Rank	**1**	6	4	2	5	9	8	3	7
F28	Best	**3.27E+03**	3.35E+03	3.45E+03	4.26E+03	3.53E+03	4.41E+03	3.61E+03	3.38E+03	3.62E+03
	Mean	**3.55E+03**	3.95E+03	3.71E+03	4.65E+03	3.90E+03	4.91E+03	3.92E+03	3.82E+03	4.13E+03
	Std	1.82E+02	2.73E+02	1.78E+02	2.11E+02	2.35E+02	2.99E+02	**1.60E+02**	2.19E+02	2.06E+02
	Rank	**1**	6	2	8	4	9	5	3	7
F29	Best	5.26E+03	5.49E+03	**4.95E+03**	1.27E+05	5.18E+03	1.32E+07	4.52E+05	5.11E+03	8.05E+03
	Mean	7.44E+03	7.86E+03	8.94E+03	6.30E+05	1.78E+04	1.00E+08	7.11E+06	**7.10E+03**	6.31E+05
	Std	**1.36E+03**	2.09E+03	3.18E+03	4.16E+05	1.09E+04	2.05E+08	7.18E+06	2.15E+03	9.22E+05
	Rank	2	3	4	7	5	9	8	**1**	6

Table 2 shows the exemplary performance of the ODO and ISGTOA algorithms on unimodal functions F1 and F2, respectively. Among the multimodal functions F3–F9, the SO algorithm exhibits commendable performance on F9, whereas ODO secures the top position. Additionally, in the case of mixed functions F10–F19, the PSO method emerges as the victor in F12, whereas the TLBO method proves to be competitive in F15 and F16. Notably, ODO continued to perform well in numerous other functions. It is noteworthy that while ODO outperforms PSO in certain aspects, such as optimal value, mean value, and variance on the F12 function, its overall ranking is inferior to that of PSO. This discrepancy arises from the fact that PSO yielded relatively poor results only once out of 30 independent runs, leading to a lower numerical performance compared to ODO. However, according to Friedman’s test, PSO is superior to ODO, highlighting the significance of employing Friedman’s test in such evaluations. Among the composite functions F20–F29, ODO demonstrated exceptional performance in six instances, whereas ILA exhibited commendable results in F25, ISGTOA excelled in F23 and F29, and FOX performed well in F24. These findings suggest that composite functions pose significant challenges for all algorithms. According to the data presented in Table 2, ODO shows notable competitiveness in multimodal and mixed functions and also displays a certain level of competitiveness in other function types.

Table 3 presents the outcomes of the Wilcoxon signed-rank test^51^ for the four dimensions of the CEC2017 test set, employing the significance level of $\alpha =0.05$. Additionally, the table includes the number of successes, similarities, and failures of ODO in comparison with the aforementioned methods, which are displayed at the bottom of the table. The symbols ‘+’, ‘$\approx$’, and ‘−’ in the table indicate the superiority, approximation, and inferiority of our proposed method compared to the comparison method, respectively, and are presented as ‘Win | Similar | Loss’ at the bottom of the table. The table presents the dimensions of the test functions 10, 20, 30, 50, and 100. Upon analyzing the comparison results, it is evident that the ODO algorithm exhibits commendable performance across all four dimensions.

As shown in Table 3, the ODO algorithm demonstrated a noteworthy enhancement in performance on low-dimensional data, as evidenced by its victory in 188 instances, 38 draws, and only 14 losses out of the total encounters on the 10-dimensional dataset in the CEC2017 test set. In the context of a dimension of 30, the ODO algorithm demonstrated a significant enhancement in performance, attaining 199 victories, 25 draws, and only eight losses against the opposing entity. Similarly, when the dimensionality increased to 50, the ODO algorithm achieved 161 victories, 41 draws, and 7 losses against the opponent. Similarly, in the scenario of a dimensionality of 100, the ODO algorithm accomplished 193 victories, 26 draws, and 13 losses against the opposing entity. The ODO algorithm demonstrated superior performance on dimension 30, followed by dimension 100, but exhibited a minor decline in other dimensions. Among the comparison methods, excluding ESO and SHO, the instances where the alternative algorithms outperformed ODO remained approximately constant at 7. In general, the ODO algorithm consistently outperformed the algorithms employed for comparison across various dimensions and functions, thereby indicating a noteworthy enhancement in performance on the CEC2017 test set.

**Table 3:** Wilcoxon’s signed rank test under the CEC2017 benchmarks.

Function	PSO	TLBO	FOX	SO	SHO	ILA	ISGTOA	ESO
	10/30/50/100	10/30/50/100	10/30/50/100	10/30/50/100	10/30/50/100	10/30/50/100	10/30/50/100	10/30/50/100
F1	+$\backslash$+$\backslash$+$\backslash$+	+$\backslash$+$\backslash$=$\backslash$+	+$\backslash$+$\backslash$=$\backslash$+	+$\backslash$+$\backslash$+$\backslash$+	+$\backslash$+$\backslash$+$\backslash$+	+$\backslash$+$\backslash$+$\backslash$+	+$\backslash$+$\backslash$=$\backslash$+	+$\backslash$+$\backslash$+$\backslash$+
F2	=$\backslash$−$\backslash$−$\backslash$−	=$\backslash$+$\backslash$−$\backslash$=	=$\backslash$−$\backslash$−$\backslash$+	=$\backslash$+$\backslash$+$\backslash$=	+$\backslash$+$\backslash$+$\backslash$=	+$\backslash$+$\backslash$+$\backslash$−	=$\backslash$−$\backslash$−$\backslash$−	+$\backslash$+$\backslash$+$\backslash$+
F3	+$\backslash$+$\backslash$+$\backslash$+	+$\backslash$+$\backslash$+$\backslash$+	+$\backslash$+$\backslash$+$\backslash$=	+$\backslash$+$\backslash$+$\backslash$+	+$\backslash$+$\backslash$+$\backslash$+	+$\backslash$+$\backslash$+$\backslash$+	=$\backslash$+$\backslash$=$\backslash$+	+$\backslash$+$\backslash$+$\backslash$+
F4	+$\backslash$+$\backslash$+$\backslash$+	=$\backslash$+$\backslash$+$\backslash$+	+$\backslash$+$\backslash$+$\backslash$+	+$\backslash$+$\backslash$=$\backslash$−	+$\backslash$+$\backslash$+$\backslash$+	+$\backslash$+$\backslash$+$\backslash$+	+$\backslash$+$\backslash$+$\backslash$=	+$\backslash$+$\backslash$+$\backslash$+
F5	+$\backslash$+$\backslash$+$\backslash$+	+$\backslash$+$\backslash$+$\backslash$+	+$\backslash$+$\backslash$+$\backslash$+	+$\backslash$+$\backslash$+$\backslash$+	+$\backslash$+$\backslash$+$\backslash$+	+$\backslash$+$\backslash$+$\backslash$+	+$\backslash$+$\backslash$+$\backslash$+	+$\backslash$+$\backslash$+$\backslash$+
F6	+$\backslash$+$\backslash$=$\backslash$−	+$\backslash$+$\backslash$+$\backslash$+	+$\backslash$+$\backslash$+$\backslash$+	+$\backslash$=$\backslash$−$\backslash$−	+$\backslash$+$\backslash$+$\backslash$+	+$\backslash$+$\backslash$+$\backslash$+	+$\backslash$+$\backslash$+$\backslash$+	+$\backslash$+$\backslash$+$\backslash$=
F7	+$\backslash$+$\backslash$+$\backslash$+	=$\backslash$+$\backslash$+$\backslash$+	+$\backslash$+$\backslash$+$\backslash$+	+$\backslash$+$\backslash$+$\backslash$−	+$\backslash$+$\backslash$+$\backslash$+	+$\backslash$+$\backslash$+$\backslash$+	+$\backslash$+$\backslash$+$\backslash$+	=$\backslash$+$\backslash$+$\backslash$+
F8	=$\backslash$+$\backslash$+$\backslash$+	+$\backslash$+$\backslash$+$\backslash$+	+$\backslash$+$\backslash$+$\backslash$+	+$\backslash$+$\backslash$+$\backslash$=	+$\backslash$+$\backslash$+$\backslash$+	+$\backslash$+$\backslash$+$\backslash$+	+$\backslash$+$\backslash$+$\backslash$+	=$\backslash$+$\backslash$+$\backslash$+
F9	+$\backslash$=$\backslash$=$\backslash$=	=$\backslash$+$\backslash$+$\backslash$+	+$\backslash$+$\backslash$=$\backslash$=	+$\backslash$=$\backslash$=$\backslash$−	+$\backslash$+$\backslash$+$\backslash$+	+$\backslash$=$\backslash$+$\backslash$=	+$\backslash$+$\backslash$=$\backslash$=	+$\backslash$=$\backslash$=$\backslash$+
F10	+$\backslash$+$\backslash$=$\backslash$+	+$\backslash$+$\backslash$+$\backslash$+	+$\backslash$+$\backslash$+$\backslash$+	+$\backslash$+$\backslash$+$\backslash$+	+$\backslash$+$\backslash$+$\backslash$+	+$\backslash$+$\backslash$+$\backslash$+	+$\backslash$+$\backslash$+$\backslash$+	+$\backslash$+$\backslash$+$\backslash$+
F11	+$\backslash$+$\backslash$+$\backslash$+	+$\backslash$+$\backslash$+$\backslash$+	+$\backslash$+$\backslash$+$\backslash$+	+$\backslash$+$\backslash$+$\backslash$+	+$\backslash$+$\backslash$+$\backslash$+	+$\backslash$+$\backslash$+$\backslash$+	+$\backslash$=$\backslash$=$\backslash$+	+$\backslash$+$\backslash$+$\backslash$+
F12	+$\backslash$=$\backslash$+$\backslash$+	+$\backslash$=$\backslash$+$\backslash$+	+$\backslash$+$\backslash$+$\backslash$+	+$\backslash$+$\backslash$+$\backslash$+	+$\backslash$+$\backslash$+$\backslash$+	+$\backslash$+$\backslash$+$\backslash$+	+$\backslash$=$\backslash$+$\backslash$+	+$\backslash$+$\backslash$+$\backslash$+
F13	+$\backslash$+$\backslash$+$\backslash$+	+$\backslash$+$\backslash$+$\backslash$+	+$\backslash$+$\backslash$+$\backslash$+	+$\backslash$+$\backslash$+$\backslash$+	+$\backslash$+$\backslash$+$\backslash$+	+$\backslash$+$\backslash$+$\backslash$+	+$\backslash$=$\backslash$=$\backslash$+	+$\backslash$+$\backslash$+$\backslash$+
F14	+$\backslash$+$\backslash$=$\backslash$=	+$\backslash$+$\backslash$=$\backslash$=	+$\backslash$+$\backslash$+$\backslash$+	+$\backslash$+$\backslash$=$\backslash$+	+$\backslash$+$\backslash$+$\backslash$+	+$\backslash$+$\backslash$+$\backslash$+	+$\backslash$+$\backslash$=$\backslash$+	+$\backslash$+$\backslash$+$\backslash$+
F15	+$\backslash$+$\backslash$=$\backslash$=	+$\backslash$=$\backslash$=$\backslash$=	+$\backslash$+$\backslash$+$\backslash$+	+$\backslash$=$\backslash$=$\backslash$=	+$\backslash$+$\backslash$+$\backslash$+	+$\backslash$+$\backslash$+$\backslash$+	+$\backslash$=$\backslash$=$\backslash$=	+$\backslash$+$\backslash$+$\backslash$+
F16	+$\backslash$+$\backslash$+$\backslash$=	+$\backslash$=$\backslash$=$\backslash$=	+$\backslash$+$\backslash$+$\backslash$+	+$\backslash$+$\backslash$+$\backslash$+	+$\backslash$+$\backslash$+$\backslash$+	+$\backslash$=$\backslash$+$\backslash$+	+$\backslash$+$\backslash$+$\backslash$+	+$\backslash$+$\backslash$+$\backslash$+
F17	+$\backslash$+$\backslash$+$\backslash$+	+$\backslash$+$\backslash$+$\backslash$+	+$\backslash$+$\backslash$+$\backslash$+	+$\backslash$+$\backslash$+$\backslash$+	+$\backslash$+$\backslash$+$\backslash$+	+$\backslash$+$\backslash$+$\backslash$+	+$\backslash$+$\backslash$+$\backslash$+	+$\backslash$+$\backslash$+$\backslash$+
F18	+$\backslash$+$\backslash$=$\backslash$+	+$\backslash$+$\backslash$=$\backslash$+	+$\backslash$+$\backslash$+$\backslash$+	+$\backslash$+$\backslash$=$\backslash$+	+$\backslash$+$\backslash$+$\backslash$+	+$\backslash$+$\backslash$+$\backslash$+	+$\backslash$+$\backslash$−$\backslash$+	+$\backslash$+$\backslash$+$\backslash$+
F19	+$\backslash$+$\backslash$+$\backslash$+	+$\backslash$+$\backslash$+$\backslash$+	+$\backslash$+$\backslash$+$\backslash$+	+$\backslash$+$\backslash$+$\backslash$=	+$\backslash$+$\backslash$+$\backslash$+	+$\backslash$+$\backslash$+$\backslash$+	+$\backslash$+$\backslash$+$\backslash$+	+$\backslash$+$\backslash$+$\backslash$+
F20	=$\backslash$+$\backslash$+$\backslash$+	−$\backslash$+$\backslash$+$\backslash$+	+$\backslash$+$\backslash$+$\backslash$+	+$\backslash$+$\backslash$+$\backslash$+	+$\backslash$+$\backslash$+$\backslash$+	−$\backslash$+$\backslash$+$\backslash$+	=$\backslash$+$\backslash$+$\backslash$+	=$\backslash$+$\backslash$+$\backslash$+
F21	=$\backslash$+$\backslash$+$\backslash$=	=$\backslash$+$\backslash$+$\backslash$+	+$\backslash$+$\backslash$+$\backslash$=	=$\backslash$+$\backslash$=$\backslash$−	+$\backslash$+$\backslash$+$\backslash$+	=$\backslash$+$\backslash$+$\backslash$+	−$\backslash$+$\backslash$+$\backslash$=	+$\backslash$+$\backslash$=$\backslash$+
F22	+$\backslash$+$\backslash$+$\backslash$+	−$\backslash$+$\backslash$+$\backslash$+	+$\backslash$+$\backslash$+$\backslash$+	+$\backslash$+$\backslash$+$\backslash$+	+$\backslash$+$\backslash$+$\backslash$+	=$\backslash$+$\backslash$+$\backslash$+	=$\backslash$+$\backslash$+$\backslash$+	−$\backslash$+$\backslash$+$\backslash$+
F23	=$\backslash$+$\backslash$+$\backslash$+	−$\backslash$=$\backslash$=$\backslash$+	+$\backslash$+$\backslash$+$\backslash$+	=$\backslash$=$\backslash$=$\backslash$+	+$\backslash$+$\backslash$+$\backslash$+	−$\backslash$=$\backslash$+$\backslash$+	=$\backslash$−$\backslash$=$\backslash$+	=$\backslash$−$\backslash$=$\backslash$+
F24	−$\backslash$=$\backslash$=$\backslash$=	=$\backslash$+$\backslash$+$\backslash$+	+$\backslash$−$\backslash$=$\backslash$−	−$\backslash$−$\backslash$=$\backslash$+	+$\backslash$+$\backslash$+$\backslash$+	−$\backslash$+$\backslash$+$\backslash$+	=$\backslash$+$\backslash$=$\backslash$+	=$\backslash$+$\backslash$+$\backslash$+
F25	+$\backslash$+$\backslash$=$\backslash$=	=$\backslash$=$\backslash$+$\backslash$+	+$\backslash$+$\backslash$+$\backslash$−	+$\backslash$+$\backslash$=$\backslash$=	+$\backslash$+$\backslash$+$\backslash$+	−$\backslash$−$\backslash$−$\backslash$−	+$\backslash$+$\backslash$=$\backslash$+	=$\backslash$+$\backslash$+$\backslash$+
F26	+$\backslash$+$\backslash$+$\backslash$+	−$\backslash$+$\backslash$+$\backslash$+	+$\backslash$+$\backslash$+$\backslash$+	=$\backslash$+$\backslash$+$\backslash$+	+$\backslash$+$\backslash$+$\backslash$+	−$\backslash$+$\backslash$+$\backslash$+	−$\backslash$=$\backslash$+$\backslash$+	=$\backslash$=$\backslash$+$\backslash$+
F27	+$\backslash$+$\backslash$+$\backslash$+	=$\backslash$+$\backslash$+$\backslash$+	+$\backslash$=$\backslash$=$\backslash$−	+$\backslash$+$\backslash$+$\backslash$+	+$\backslash$+$\backslash$+$\backslash$+	=$\backslash$+$\backslash$+$\backslash$+	+$\backslash$+$\backslash$+$\backslash$+	+$\backslash$+$\backslash$+$\backslash$+
F28	+$\backslash$+$\backslash$+$\backslash$+	=$\backslash$+$\backslash$+$\backslash$+	+$\backslash$+$\backslash$+$\backslash$+	+$\backslash$+$\backslash$+$\backslash$+	+$\backslash$+$\backslash$+$\backslash$+	=$\backslash$+$\backslash$+$\backslash$+	=$\backslash$+$\backslash$+$\backslash$+	=$\backslash$+$\backslash$+$\backslash$+
F29	+$\backslash$=$\backslash$=$\backslash$+	+$\backslash$+$\backslash$+$\backslash$+	+$\backslash$+$\backslash$+$\backslash$+	+$\backslash$+$\backslash$+$\backslash$+	+$\backslash$+$\backslash$+$\backslash$+	+$\backslash$+$\backslash$+$\backslash$+	+$\backslash$=$\backslash$+$\backslash$+	+$\backslash$+$\backslash$+$\backslash$+
Wilcoxon-10	23 | 5 | 1	16 | 9 | 4	28 | 1 | 0	24 | 4 | 1	29 | 0 | 0	20 | 4 | 5	20 | 7 | 2	20 | 8 | 1
Wilcoxon-30	24 | 4 | 1	24 | 5 | 0	26 | 1 | 2	24 | 4 | 1	29 | 0 | 0	25 | 3 | 1	21 | 6 | 2	26 | 2 | 1
Wilcoxon-50	19 | 9 | 1	22 | 6 | 1	24 | 4 | 1	19 | 9 | 1	29 | 0 | 0	28 | 0 | 1	17 | 10 | 2	26 | 3 | 0
Wilcoxon-100	20 | 7 | 2	25 | 4 | 0	23 | 3 | 3	19 | 5 | 5	28 | 1 | 0	26 | 1 | 2	24 | 4 | 1	28 | 1 | 0

In conjunction with the CEC2017 test set, the CEC2022 test set was employed to evaluate the efficacy of the ODO algorithm. The results of the Wilcoxon signed-rank test, in comparison with alternative algorithms, are presented in Table 4. Specifically, on the CEC2022 test set, out of a total of 192 comparisons, the ODO algorithm achieved 139 victories, while there were 45 instances of comparable performance and only eight occurrences of inferiority to its adversaries. These results demonstrate the commendable performance of the ODO algorithm on the CEC2022 test set.

**Table 4:** Wilcoxon’s signed rank test under the CEC2022 benchmarks.

Function	PSO	TLBO	FOX	SO	SHO	ILA	ISGTOA	ESO
	10/20	10/20	10/20	10/20	10/20	10/20	10/20	10/20
F30	=$\backslash$=	=$\backslash$=	=$\backslash$+	=$\backslash$+	+$\backslash$+	+$\backslash$+	=$\backslash$=	+$\backslash$+
F31	+$\backslash$+	+$\backslash$+	+$\backslash$+	+$\backslash$+	+$\backslash$+	+$\backslash$+	+$\backslash$=	=$\backslash$+
F32	+$\backslash$+	+$\backslash$+	+$\backslash$+	+$\backslash$+	+$\backslash$+	+$\backslash$+	+$\backslash$+	+$\backslash$+
F33	+$\backslash$+	−$\backslash$=	+$\backslash$+	+$\backslash$=	+$\backslash$+	=$\backslash$+	=$\backslash$=	−$\backslash$=
F34	−$\backslash$+	=$\backslash$=	+$\backslash$+	=$\backslash$+	+$\backslash$+	=$\backslash$+	=$\backslash$=	−$\backslash$=
F35	+$\backslash$+	+$\backslash$+	+$\backslash$+	+$\backslash$+	+$\backslash$+	+$\backslash$+	+$\backslash$+	+$\backslash$+
F36	+$\backslash$+	+$\backslash$+	+$\backslash$+	+$\backslash$+	+$\backslash$+	+$\backslash$+	+$\backslash$+	+$\backslash$+
F37	+$\backslash$+	+$\backslash$+	+$\backslash$+	+$\backslash$+	+$\backslash$+	+$\backslash$+	=$\backslash$+	+$\backslash$+
F38	+$\backslash$+	+$\backslash$+	+$\backslash$+	+$\backslash$+	+$\backslash$+	+$\backslash$+	+$\backslash$+	+$\backslash$+
F39	+$\backslash$+	=$\backslash$+	+$\backslash$+	+$\backslash$+	+$\backslash$+	=$\backslash$+	=$\backslash$+	=$\backslash$+
F40	=$\backslash$=	=$\backslash$=	=$\backslash$+	=$\backslash$=	+$\backslash$+	=$\backslash$+	=$\backslash$=	=$\backslash$=
F41	+$\backslash$+	−$\backslash$=	+$\backslash$+	=$\backslash$+	+$\backslash$+	−$\backslash$=	−$\backslash$=	−$\backslash$=
Wilcoxon-10	9 | 2 | 1	6 | 4 | 2	10 | 2 | 0	8 | 4 | 0	12 | 0 | 0	7 | 4 | 1	5 | 6 | 1	6 | 3 | 3
Wilcoxon-20	10 | 2 | 0	7 | 5 | 0	12 | 0 | 0	10 | 2 | 0	12 | 0 | 0	11 | 1 | 0	6 | 6 | 0	8 | 4 | 0

**Fig. 4:**
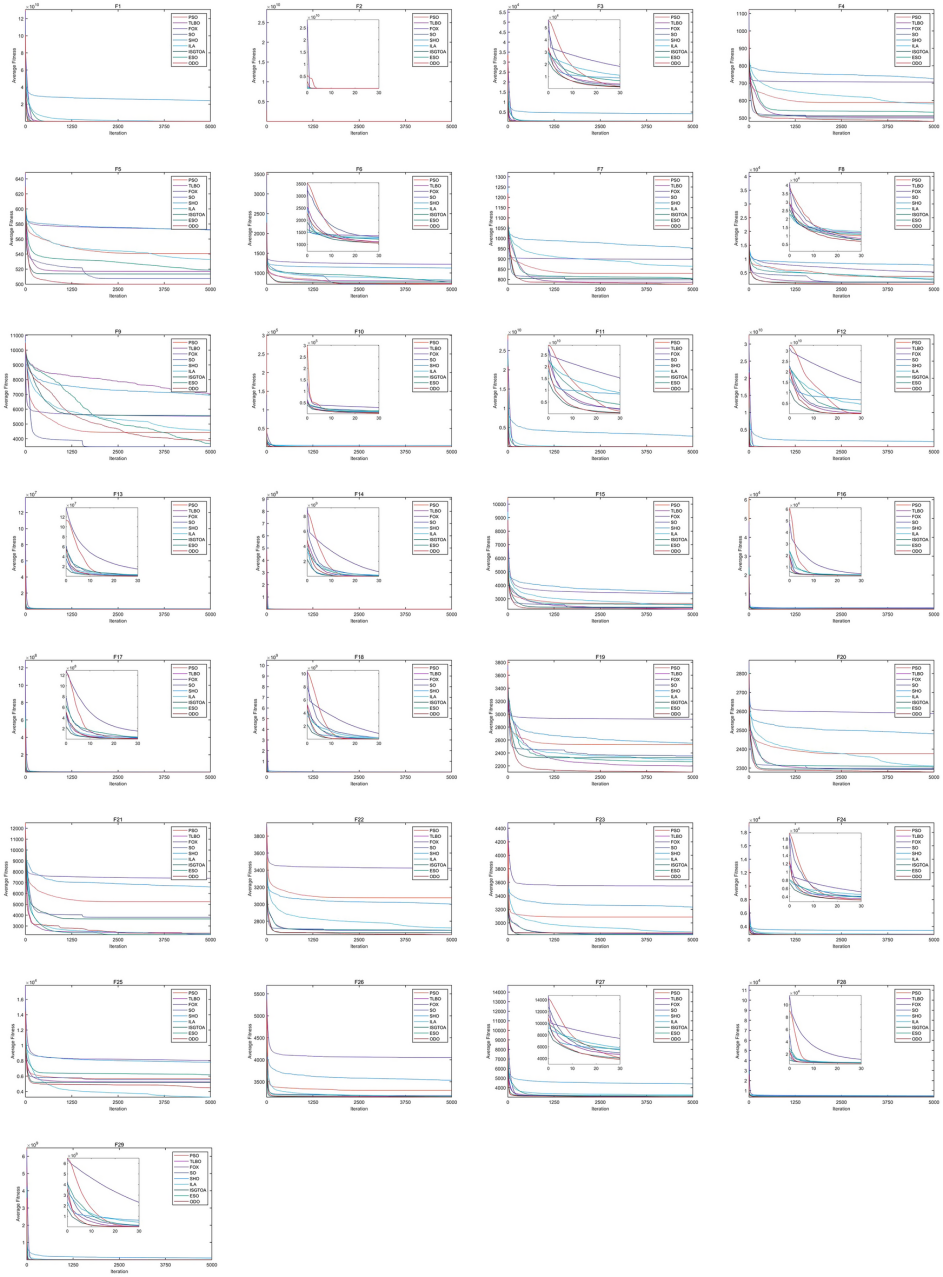
The convergence curves of ODO and the other 8 metaheuristic algorithms on CEC2017 test suit with dimension of 30.

Figure 4 presents the convergence curve of the average optimal solution for ODO and other algorithms observed over 5000 iterations on the 30 dimensions of the CEC2017 test set. The graph demonstrates that the ODO algorithm consistently displays favorable convergence traits throughout the entire iteration process, surpassing the majority of the methods in terms of convergence speed. Moreover, it exhibits a commendable capability to escape local minima, thereby progressively attaining superior functional solutions. During the iterative process, the algorithms exhibited a similar trajectory in achieving the global optimal solution, characterized by a consistent exponential descent. Within the initial 300 iterations, a rapid descent is observed, leading to a relatively stable stage. Subsequently, the algorithms progressively converged towards the optimal solution of the function, relying on their respective capabilities to escape local minima. The convergence curve clearly demonstrates the efficacy of each algorithm in escaping the local minima. Notably, starting from function F3, the discrepancy in the heights between the curves became pronounced. In general, the ODO algorithm exhibits consistent convergence across unimodal, multimodal, mixed, and composite functions, all of which display gradual descent. This observation suggests that the ODO algorithm possesses a certain level of versatility, rendering it capable of effectively addressing a wide range of functional optimization problems.

In conclusion, the ODO algorithm demonstrated significant competitiveness in effectively addressing unimodal, multimodal, mixed, and composite functions, thereby exhibiting a commendable overall performance in terms of optimization accuracy.

### Engineering case studies to test ODO

This section introduces two practical applications, namely pressure vessel design and gas transmission compressor problems, which need to be optimized through the utilization of ODO. The constraints in the two applications are processed using Eqs. (4)–(6) and the penalty coefficient is assigned a value of ${{10}^{20}}$.

#### Solving a pressure vessel design problem

**Fig. 5:**
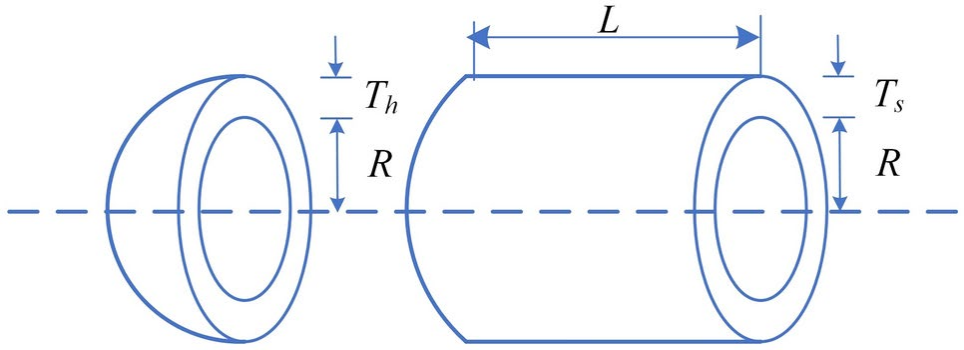
Schematic view of the pressure vessel design problem.

The problem, initially introduced by Kannan and Kramer (1994), has been addressed by various researchers, who have employed diverse algorithms to find solutions^33,47^. The objective of this problem is to minimize the overall cost of a pressure vessel, shown in Fig. 5, considering the expenses associated with material, forming, and welding. The problem incorporates four design variables: shell thickness ${{T}_{s}}({{x}_{1}})$, head thickness ${{T}_{h}}({{x}_{2}})$, inner radius $R({{x}_{3}})$, and length of the cylindrical section $L({{x}_{4}})$ of the vessel. The mathematical formulation of this problem is expressed as follows:

23 $$\begin{aligned} \begin{aligned} \min f(x)&=0.6224{{x}_{1}}{{x}_{3}}{{x}_{4}}+1.7781{{x}_{2}}x_{3}^{2}+3.1661x_{1}^{2}{{x}_{4}}+19.84x_{1}^{2}{{x}_{3}} \\ s.t.\,{{g}_{1}}(x)&=-{{x}_{1}}+0.0193{{x}_{3}}\le 0 \\ {{g}_{2}}(x)&=-{{x}_{2}}+0.00954{{x}_{3}}\le 0 \\ {{g}_{3}}(x)&=-\pi x_{3}^{2}{{x}_{4}}-(4/3)\pi x_{3}^{2}+1296000\le 0 \\ {{g}_{4}}(x)&={{x}_{4}}-240\le 0 \end{aligned} \end{aligned}$$

where the decision variables ${{x}_{1}}$, ${{x}_{2}}$, ${{x}_{3}}$, ${{x}_{4}}$ are continuous, with bounds: $0\le {{x}_{1}}\le 99,0\le {{x}_{2}}\le 99,10\le {{x}_{3}}\le 200,10\le {{x}_{4}}\le 200$.

To establish the superior performance of ODO in comparison with other algorithms, the utilization of ODO to address this problem is undertaken. The findings presented in Table 5 demonstrate the notable efficiency of ODO compared to alternative algorithms. Collectively, the successful resolution of this problem provides further evidence of the highly competitive nature of ODO.

**Table 5:** Comparison for the pressure vessel design problem. Significant values are in bold.

Algorithm	${{x}_{1}}$	${{x}_{2}}$	${{x}_{3}}$	${{x}_{4}}$	Best
ODO	7.78E−01	3.85E−01	4.03E+01	2.00E+02	**5.89E+03**
PSO	7.78E−01	3.85E−01	4.03E+01	2.00E+02	**5.89E+03**
TLBO	7.78E−01	3.85E−01	4.03E+01	2.00E+02	**5.89E+03**
FOX	7.86E−01	3.89E−01	4.07E+01	1.94E+02	5.90E+03
SO	7.78E−01	3.85E−01	4.03E+01	2.00E+02	5.89E+03
SHO	7.83E−01	3.87E−01	4.05E+01	1.97E+02	5.89E+03
ILA	7.78E−01	3.85E−01	4.03E+01	2.00E+02	5.89E+03
ISGTOA	7.78E−01	3.85E−01	4.03E+01	2.00E+02	**5.89E+03**
ESO	7.78E−01	3.85E−01	4.03E+01	2.00E+02	**5.89E+03**

#### Solving a gas transmission compressor problem

The optimization of the gas transmission compressor design involves four design variables and one constraint condition^33^. The mathematical model for this problem is as follows:

24 $$\begin{aligned} \begin{aligned} \min&f(x)=8.61\times {{10}^{5}}x_{1}^{1/2}{{x}_{2}}x_{3}^{-2/3}x_{4}^{-1/2}+3.69\times {{10}^{4}}{{x}_{3}}+7.72\times {{10}^{8}}x_{1}^{-1}x_{2}^{0.219}-765.43\times {{10}^{6}}x_{1}^{-1}\\ s.t.&g(x)={{x}_{4}}x_{2}^{-2}+x_{2}^{-2}-1\le 0 \end{aligned} \end{aligned}$$

where $$20\le {{x}_{1}}\le 50;1\le {{x}_{2}}\le 10;20\le {{x}_{3}}\le 50,0.1\le {{x}_{4}}\le 60$$ .

Table 6 presents the outcomes pertaining to the gas transmission compressor problem, wherein it is observed that the ODO algorithm exhibits superior efficiency compared with the other algorithms.

**Table 6:** Comparison for the gas transmission compressor problem. Significant values are in bold.

Algorithm	${{x}_{1}}$	${{x}_{2}}$	${{x}_{3}}$	${{x}_{4}}$	Best
ODO	5.00E+01	1.18E+00	2.46E+01	3.88E−01	**2.96E+06**
PSO	5.00E+01	1.18E+00	2.46E+01	3.88E−01	2.96E+06
TLBO	5.00E+01	1.18E+00	2.46E+01	3.88E−01	2.96E+06
FOX	4.61E+01	1.16E+00	2.64E+01	3.46E−01	2.97E+06
SO	5.00E+01	1.18E+00	2.46E+01	3.88E−01	2.96E+06
SHO	2.93E+01	1.09E+00	2.51E+01	1.81E−01	3.02E+06
ILA	5.00E+01	1.18E+00	2.46E+01	3.88E−01	2.96E+06
ISGTOA	5.00E+01	1.18E+00	2.46E+01	3.88E−01	**2.96E+06**
ESO	5.00E+01	1.18E+00	2.46E+01	3.89E−01	2.96E+06

### Parameter sensitivity

The ODO algorithm comprises four control parameters that significantly influence its performance: ${{p}_{1}}$ which is offensive selection probability, ${{p}_{2}}$ which is the probability of remaining or moving for some dimension of an individual, ${{p}_{3}}$ which is the reset defense dimension selection probability, and ${{p}_{4}}$ which is the probability of selecting reset defensive. In this section, basic statistical techniques are employed to ascertain the values of these parameters and to evaluate the algorithm’s effectiveness through the selection of specific optimization functions. These functions are derived from the CEC2017 test suite, with a focus on unimodal function F1, multimodal function F4, mixed function F10, and composite function F20. The sensitivity of these parameters was determined by examining their influence on the convergence under different dimensions, including $D=10$, $D=30$, $D=50$, and $D=100$, as provided by the CEC2017 test suite. The remaining parameters remain unchanged from the previous configuration. In light of the diverse levels of impact exerted by dimensions and functional types on function optimization, Eqs. (25)–(27) are employed to standardize the errors in each optimization outcome and aggregate a cumulative sum of errors. This outcome can be utilized to determine suitable parameter values.

25 $$\begin{aligned}&TE(v)=\sum \limits _{i=1}^{{{f}_{N}}}{\sum \limits _{j=1}^{{{D}_{N}}}{\Delta {{f}_{i,j}}(v)/\Delta f_{i,j}^{\max }}}\, \end{aligned}$$

26 $$\begin{aligned}&\Delta {{f}_{i,j}}(v)=\left( \frac{1}{{{N}_{run}}}\sum \limits _{k=1}^{{{N}_{run}}}{{{f}_{i,j,k}}(v)} \right) -f_{i}^{*} \end{aligned}$$

27 $$\begin{aligned}&\Delta f_{i,j}^{\max }=\max (\Delta {{f}_{i,j}}(v)) \end{aligned}$$

where *v* denotes the parameter value; $\Delta {{f}_{i,j}}(v)$ denotes the discrepancy between the average optimal solution and the optimal solution of the function after 30 runs with the *v* parameter; $f_{i}^{*}$ denotes the optimal solution of the *i*th function; and $\Delta f_{i,j}^{\max }$ represents the maximum discrepancy obtained under varying *v* values.

Table 7 displays the total deviation of parameter ${{p}_{1}}$ at different values. Based on these findings, it is evident that ${{p}_{1}}$ achieved the lowest normalization error at values of 0.7 and 0.4, and characteristics. This suggests that the selection of the attack mode is contingent on the specific attributes of the processed function. Given the similarity between the results obtained at values 0.7 and 0.8, this study opts for 0.7 as the parameter ${{p}_{1}}$ value.

**Table 7:** Normalized error sum for four types of functions with different values of ${{p}_{1}}$.

	$${{p}_{1}}$$									
	0.05	0.1	0.2	0.3	0.4	0.5	0.6	0.7	0.8	0.9
Normalized error sum	8.57	8.8	9	9.4	8.4	8.9	8.8	8.4	8.5	14

Table 8 presents the comprehensive variations in ${{p}_{2}}$ across various values. The findings indicate that the smallest deviation occurs when ${{p}_{2}}$ is set to 0.3, with the results of the other values exhibiting no substantial deviation from this minimum. Notably, a relatively large error was observed only when ${{p}_{2}}$ was assigned a value of 0.9, suggesting that the impact of the ${{p}_{2}}$ value was relatively minor and lacked significant sensitivity.

**Table 8:** Normalized error sum for four types of functions with different values of ${{p}_{2}}$.

	$${{p}_{2}}$$									
	0.05	0.1	0.2	0.3	0.4	0.5	0.6	0.7	0.8	0.9
Normalized error sum	8.15	8.6	9.1	8.1	8.2	9.3	8.4	8.9	9	14

Table 9 presents the comprehensive deviation of parameter ${{p}_{3}}$ across various values. The findings indicate that ${{p}_{3}}$ attains a minimum normalization error of 0.1. ${{p}_{3}}$ serves as a reset dimension selection probability designed to enhance population diversity and prevent the algorithm from converging to local minima. The test outcomes further underscored the significance of the reset operation. Table 10 presents the comprehensive deviation of parameter ${{p}_{4}}$ across various values. The findings demonstrate that the minimum deviation occurs when ${{p}_{4}}$ is set at 0.01, underscoring the significance of the reset defense. It is crucial to avoid excessively large or small values for optimal outcome.

**Table 9:** Normalized error sum for four types of functions with different values of ${{p}_{3}}$.

	$${{p}_{3}}$$									
	0.05	0.1	0.2	0.3	0.4	0.5	0.6	0.7	0.8	0.9
Normalized error sum	8.4	8.26	8.5	14	8.2	8.8	9.2	9.4	8.7	9.1

**Table 10:** Normalized error sum for four types of functions with different values of ${{p}_{4}}$.

	$${{p}_{4}}$$			
	0.0001	0.001	0.01	0.1
Normalized error sum	10.712	11.03	9.7	12

### Diversity of population

**Fig. 6:**
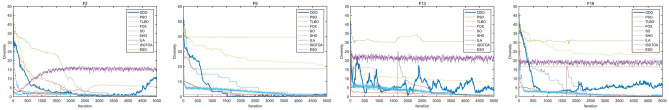
Diversity behavior for F2, F9, F13 and F19 of CEC2017.

Four functions, F2, F9, F13, and F19, from the CEC2017 benchmark suite are utilized to illustrate the relationship between population diversity and the number of iterations^52^. As depicted in Fig. 6, it is evident that numerous algorithms enhance population diversity in specific ways throughout the search process. The FOX algorithm adopts a strategy aimed at preserving fundamental diversity levels. SO algorithm parallels FOX algorithm in its necessity for diversity when addressing more complex optimization functions, while it incrementally diminishes this requirement for relatively simpler functions, such as F2. In contrast, the ILA algorithm intermittently introduces substantial diversity during the convergence process of population diversity, thereby exhibiting quadratic convergence behavior. The proposed ODO algorithm demonstrates convergence properties in its general trajectory, although specific details may differ depending on the particular problem being addressed. For instance, in the F2 problem, ODO algorithm enhances diversity towards the end of the iteration. It maintains a continuous convergence state in F9, exhibits oscillatory behavior in F13, and increases diversity during the intermediate and later stages in F19. These variations in diversity and convergence are advantageous for identifying the optimal solution, as corroborated by the results presented in Table 2.

**Fig. 7:**
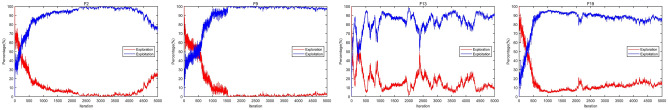
Exploration and exploitation of ODO on F2, F9, F13 and F19 of CEC2017.

Figure 7 illustrates the exploration and exploitation performance of the ODO algorithm in the context of solving functions F2, F9, F13, and F19^53^. The graph reveals that the algorithm’s exploration capability progressively enhances from an initial state of weakness to become its primary search mechanism, whereas the exploitation capability exhibits an inverse trend. In comparison to Fig. 6, these observations are further corroborated by corresponding changes in population diversity, which are also indicative of the algorithm’s exploration and exploitation dynamics.

### Time consumption

This section undertakes a comparative analysis of the time complexity associated with the algorithms under consideration. For the algorithms PSO, FOX, SO, and ESO, the time complexity exhibits a linear increase with respect to population size *N*, dimension *D*, and the number of iterations ${T_{\text {max}}}$, expressed as ${\mathrm O}(D\times N\times {{T}_{\max }})$. In contrast, the time complexities for the TLBO, SHO, and ISGTOA algorithms are ${\mathrm O}(D\times 2N\times {{T}_{\max }})$, ${\mathrm O}(D\times 3N\times {{T}_{\max }})$, and ${\mathrm O}(D\times 2N\times {{T}_{\max }})$, respectively. It is noteworthy that the population size *N* increases by varying multiples under consistent iteration counts and dimensional settings. The ILA algorithm’s time complexity is characterized by ${\mathrm O}(D\times {N}^{\beta }\times {{T}_{\max }})$, where ${\beta >1}$; the precise value of ${\beta }$ is contingent upon specific algorithm parameters. The ODO algorithm demonstrates a time complexity of ${\mathrm O}(D\times N\times ({{T}_{\max }}+1))$, which, relative to the other algorithms, does not significantly augment the overall time complexity.

**Fig. 8:**
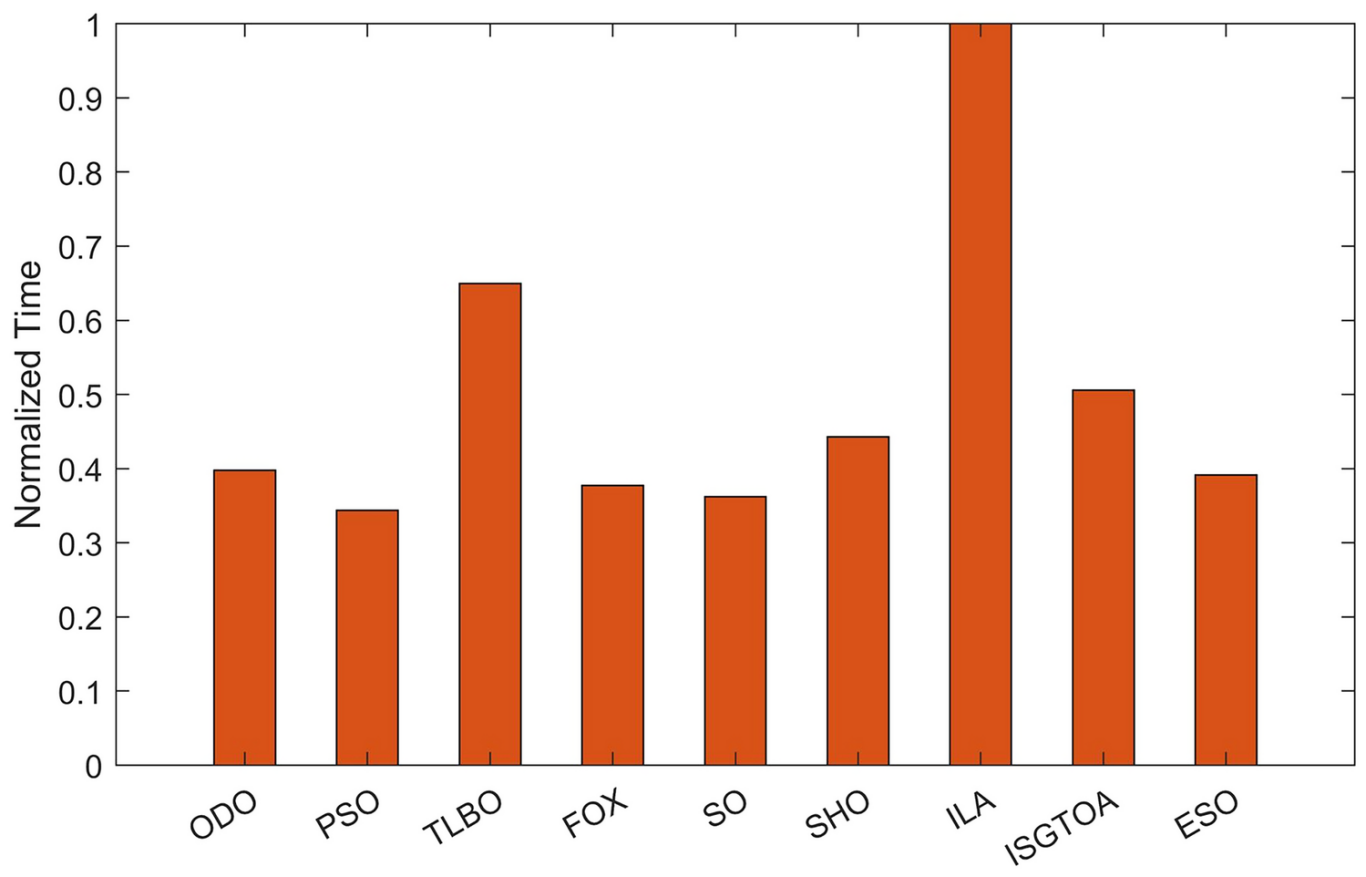
Bar plot of the time consumptions under CEC2017 test suit.

For enhanced clarity, comparisons were conducted under the condition of an identical maximum number of function evaluations. The total computational time required for the 29 benchmark functions (F1–F29) within the CEC2017 test suite was compared and normalized against the maximum observed time consumption, as depicted in Fig. 8. Analysis of the graph indicates that the time consumption of the ODO algorithm is comparable to that of most other algorithms and is significantly lower than that of the ILA algorithm. While the ODO algorithm did not demonstrate notably competitive performance regarding computational complexity, it achieved superior results within the same computational time frame, as illustrated in Table 2.

## Conclusion

In this study, a novel metaheuristic algorithm called ODO, which draws inspiration from board games, was introduced to address continuous optimization problems. The design of the algorithm is primarily motivated by the strategic aspects of attacking and defending in the game. The decision-making process of a player is influenced by various factors, including the spatial arrangement of the pieces on the board, characteristics of the pieces themselves, and the chosen confrontation strategy. By incorporating these principles and phenomena into the search behaviors employed during the optimization process, a hybrid search behavior was devised to augment the search capability of ODO for continuous optimization problems. This study aimed to assess the efficacy and efficiency of ODO by employing two CEC benchmarks, namely CEC2017 and CEC2022, along with two engineering concerns. The simulation outcomes indicate that ODO demonstrated enhanced performance in a significant majority of the examined optimization problems. Although ODO performs well on most tests, its weaker results in certain cases suggest areas for improvement, particularly in strengthening the optimization framework and refining particle movement strategies. These limitations guide our current efforts, which focus on developing a dynamic movement strategy to balance exploration and exploitation and expanding ODO to handle binary and multi-objective optimization problems, thereby improving the algorithm’s capabilities and applications.

## Data Availability

All data generated or analyzed during this study are available from the corresponding author.

## References

[1] Deaconu, A. M., Cotfas, D. T. & Cotfas, P. A. Advanced optimization methods and applications. *Mathematics* **11**, 2205–2211. 10.3390/math11092205 (2023).

[2] Akyol, S. & Alatas, B. Plant intelligence based metaheuristic optimization algorithms. *Artif. Intell. Rev.* **47**, 417–462. 10.1007/s10462-016-9486-6 (2017).

[3] Goldberg, D. E. *Genetic Algorithms in Search, Optimization, and Machine Learning* (Addison-Wesley Pub. Co, 1989).

[4] Storn, R. Differential evolution—A simple and efficient heuristic for global optimization over continuous spaces. *J. Glob. Optim.* **11**, 341–359. 10.1023/A:1008202821328 (1997).

[5] Kennedy, J. & Eberhart, R. Particle swarm optimization. In *Proceedings of ICNN’95—International Conference on Neural Networks*, vol. 4, 1942–1948. 10.1109/ICNN.1995.488968 (IEEE, 1995).

[6] Gandomi, A. H., Yang, X.-S., Alavi, A. H. & Talatahari, S. Bat algorithm for constrained optimization tasks. *Neural Comput. Appl.* **22**, 1239–1255. 10.1007/s00521-012-1028-9 (2013).

[7] Geem, Z. W., Kim, J. H. & Loganathan, G. A new heuristic optimization algorithm: Harmony search. *Simulation* **76**, 60–68. 10.1177/003754970107600201 (2001).

[8] Karaboga, D. & Basturk, B. On the performance of artificial bee colony (ABC) algorithm. *Appl. Soft Comput.* **8**, 687–697. 10.1016/j.asoc.2007.05.007 (2008).

[9] Li, M., Liu, Z. & Song, H. An improved algorithm optimization algorithm based on RungeKutta and golden sine strategy. *Expert Syst. Appl.* **247**, 123262–123278. 10.1016/j.eswa.2024.123262 (2024).

[10] Duankhan, P., Sunat, K., Chiewchanwattana, S. & Nasa-ngium, P. The Differentiated Creative Search (DCS): Leveraging differentiated knowledge-acquisition and creative realism to address complex optimization problems. *Expert Syst. Appl.* **252**, 123734–123784. 10.1016/j.eswa.2024.123734 (2024).

[11] Lian, J. & Hui, G. Human evolutionary optimization algorithm. *Expert Syst. Appl.* **241**, 122638–122662. 10.1016/j.eswa.2023.122638 (2024).

[12] Ghodbane, H., Amar, H., Amir, M., Babes, B. & Hamouda, N. Biometric identification of hand by particle swarm optimization (PSO) algorithm. In *Intelligent and Fuzzy Systems* Vol. 758 (eds Kahraman, C. et al.) 454–461 (Springer Nature Switzerland, 2023). 10.1007/978-3-031-39774-5_51.

[13] Okoji, A. I., Okoji, C. N. & Awarun, O. S. Performance evaluation of artificial intelligence with particle swarm optimization (PSO) to predict treatment water plant DBPs (haloacetic acids). *Chemosphere* **344**, 140238–140252. 10.1016/j.chemosphere.2023.140238 (2023). 37788747 10.1016/j.chemosphere.2023.140238

[14] Singh, S. K., Kumar, M. & Singh, J. Integration of Particle Swarm Optimization (PSO) and machine learning to improve classification accuracy during antenna design. *Trans. Electr. Electron. Mater.* **24**, 258–266. 10.1007/s42341-023-00443-x (2023).

[15] Mammeri, E. et al. A global MPPT controller based on an improved particle swarm optimization algorithm. In *Advanced Computational Techniques for Renewable Energy Systems* Vol. 591 (ed. Hatti, M.) 281–288 (Springer International Publishing, 2023). 10.1007/978-3-031-21216-1_30.

[16] Rajesh Kumar, K. & Vijayakumar, M. Optimization of cognitive femtocell network via oppositional beetle swarm optimization algorithm. *Intell. Autom. Soft Comput.* **36**, 819–832. 10.32604/iasc.2023.030961 (2023).

[17] Kankılıç, S. & Karpat, E. Optimization of multilayer absorbers using the bald eagle optimization algorithm. *Appl. Sci.* **13**, 10301–10317. 10.3390/app131810301 (2023).

[18] Eirgash, M. A., Toğan, V. & Dede, T. Time-cost trade-off problems with multi-objective quasi-oppositional teaching learning-based optimization. In *Advanced Engineering Optimization Through Intelligent Techniques* (eds Venkata Rao, R. & Taler, J.) 269–277 (Springer Nature Singapore, 2023). 10.1007/978-981-19-9285-8_26.

[19] Brajević, I. A shuffle-based artificial bee colony algorithm for solving integer programming and minimax problems. *Mathematics* **9**, 1211. 10.3390/math9111211 (2021).

[20] Li, Y., Yuan, Q., Han, M. & Cui, R. Hybrid multi-strategy improved wild horse optimizer. *Adv. Intell. Syst.* **4**, 2200097. 10.1002/aisy.202200097 (2022).

[21] Kumari, M., De, P. K., Chaudhuri, K. & Narang, P. Utilizing a hybrid metaheuristic algorithm to solve capacitated vehicle routing problem. *Results Control Optim.* **13**, 100292. 10.1016/j.rico.2023.100292 (2023).

[22] Trojovský, P. & Dehghani, M. Pelican optimization algorithm: A novel nature-inspired algorithm for engineering applications. *Sensors* **22**, 855–888. 10.3390/s22030855 (2022). 35161600 10.3390/s22030855 PMC8838090

[23] Mohapatra, S. & Mohapatra, P. American zebra optimization algorithm for global optimization problems. *Sci. Rep.* **13**, 5211–5261. 10.1038/s41598-023-31876-2 (2023). 36997597 10.1038/s41598-023-31876-2 PMC10063666

[24] Dehghani, M., Trojovský, P. & Malik, O. P. Green anaconda optimization: A new bio-inspired metaheuristic algorithm for solving optimization problems. *Biomimetics* **8**, 121–180. 10.3390/biomimetics8010121 (2023). 36975351 10.3390/biomimetics8010121 PMC10046581

[25] Luo, K. Water flow optimizer: A nature-inspired evolutionary algorithm for global optimization. *IEEE Trans. Cybern.* **52**, 7753–7764. 10.1109/TCYB.2021.3049607 (2022). 33566779 10.1109/TCYB.2021.3049607

[26] Mirrashid, M. & Naderpour, H. Transit search: An optimization algorithm based on exoplanet exploration. *Results Control Optim.* **7**, 100127–100163. 10.1016/j.rico.2022.100127 (2022).

[27] Su, H. et al. RIME: A physics-based optimization. *Neurocomputing* **532**, 183–214. 10.1016/j.neucom.2023.02.010 (2023).

[28] Dalirinia, E., Jalali, M., Yaghoobi, M. & Tabatabaee, H. Lotus effect optimization algorithm (LEA): A lotus nature-inspired algorithm for engineering design optimization. *J. Supercomput.* **80**, 761–799. 10.1007/s11227-023-05513-8 (2023).

[29] Kaveh, M., Mesgari, M. S. & Saeidian, B. Orchard Algorithm (OA): A new meta-heuristic algorithm for solving discrete and continuous optimization problems. *Math. Comput. Simul.* **208**, 95–135. 10.1016/j.matcom.2022.12.027 (2023).

[30] Braik, M., Ryalat, M. H. & Al-Zoubi, H. A novel meta-heuristic algorithm for solving numerical optimization problems: Ali Baba and the forty thieves. *Neural Comput. Appl.* **34**, 409–455. 10.1007/s00521-021-06392-x (2022).

[31] Ma, B., Hu, Y., Lu, P. & Liu, Y. Running city game optimizer: A game-based metaheuristic optimization algorithm for global optimization. *J. Comput. Design Eng.* **10**, 65–107. 10.1093/jcde/qwac131 (2023).

[32] Zhang, W., Pan, K., Li, S. & Wang, Y. Special Forces Algorithm: A novel meta-heuristic method for global optimization. *Math. Comput. Simul.* **213**, 394–417. 10.1016/j.matcom.2023.06.015 (2023).

[33] Rao, R., Savsani, V. & Vakharia, D. Teaching-learning-based optimization: A novel method for constrained mechanical design optimization problems. *Computer-Aided Design* **43**, 303–315. 10.1016/j.cad.2010.12.015 (2011).

[34] Akbari, E., Ghasemi, M., Gil, M., Rahimnejad, A. & Andrew Gadsden, S. Optimal power flow via teaching-learning-studying-based optimization algorithm. *Electr. Power Compon. Syst.* **49**, 584–601. 10.1080/15325008.2021.1971331 (2021).

[35] Wu, D., Wang, S., Liu, Q., Abualigah, L. & Jia, H. An improved teaching-learning-based optimization algorithm with reinforcement learning strategy for solving optimization problems. *Comput. Intell. Neurosci.* **2022**, 1–24. 10.1155/2022/1535957 (2022). 10.1155/2022/1535957 PMC8970903 35371212

[36] Das, B., Mukherjee, V. & Das, D. Student psychology based optimization algorithm: A new population based optimization algorithm for solving optimization problems. *Adv. Eng. Softw.* **146**, 102804–102820. 10.1016/j.advengsoft.2020.102804 (2020).

[37] Zhang, Y. & Jin, Z. Group teaching optimization algorithm: A novel metaheuristic method for solving global optimization problems. *Expert Syst. Appl.* **148**, 113246–113263. 10.1016/j.eswa.2020.113246 (2020).

[38] Zhang, Y. & Chi, A. Group teaching optimization algorithm with information sharing for numerical optimization and engineering optimization. *J. Intell. Manuf.* **34**, 1547–1571. 10.1007/s10845-021-01872-2 (2023).

[39] Dehghani, M., Trojovská, E. & Trojovský, P. A new human-based metaheuristic algorithm for solving optimization problems on the base of simulation of driving training process. *Sci. Rep.* **12**, 9924–9944. 10.1038/s41598-022-14225-7 (2022). 35705720 10.1038/s41598-022-14225-7 PMC9200810

[40] Trojovská, E. & Dehghani, M. A new human-based metahurestic optimization method based on mimicking cooking training. *Sci. Rep.* **12**, 14861–14884. 10.1038/s41598-022-19313-2 (2022). 36050468 10.1038/s41598-022-19313-2 PMC9437068

[41] Trojovský Mohammad Dehghani, P. & Trojovský Eva Milkova, E. Language education optimization: A new human-based metaheuristic algorithm for solving optimization problems. *Comput. Model. Eng. Sci.* **136**, 1527–1573. 10.32604/cmes.2023.025908 (2023).

[42] Givi, H. & Hubalovska, M. Skill optimization algorithm: A new human-based metaheuristic technique. *Comput. Mater. Contin.* **74**, 179–202. 10.32604/cmc.2023.030379 (2023).

[43] Mirrashid, M. & Naderpour, H. Incomprehensible but intelligible-in-time logics: Theory and optimization algorithm. *Knowl.-Based Syst.* **264**, 110305–110326. 10.1016/j.knosys.2023.110305 (2023).

[44] Trojovský Mohammad Dehghani, P. Migration algorithm: A new human-based metaheuristic approach for solving optimization problems. *Comput. Model. Eng. Sci.* **137**, 1695–1730. 10.32604/cmes.2023.028314 (2023).

[45] Zolfi, K. Gold rush optimizer: A new population-based metaheuristic algorithm. *Oper. Res. Decis.* **33**, 113–150. 10.37190/ord230108 (2023).

[46] Naik, A. & Satapathy, S. C. Past present future: A new human-based algorithm for stochastic optimization. *Soft Comput.* **25**, 12915–12976. 10.1007/s00500-021-06229-8 (2021).

[47] Mohammed, H. & Rashid, T. F. O. X. A FOX-inspired optimization algorithm. *Appl. Intell.* **53**, 1030–1050. 10.1007/s10489-022-03533-0 (2023).

[48] Hashim, F. A. & Hussien, A. G. Snake Optimizer: A novel meta-heuristic optimization algorithm. *Knowl.-Based Syst.* **242**, 108320–108353. 10.1016/j.knosys.2022.108320 (2022).

[49] Zhao, S., Zhang, T., Ma, S. & Wang, M. Sea-horse optimizer: A novel nature-inspired meta-heuristic for global optimization problems. *Appl. Intell.* **53**, 11833–11860. 10.1007/s10489-022-03994-3 (2023).

[50] Yao, L. et al. ESO: An enhanced snake optimizer for real-world engineering problems. *Expert Syst. Appl.* **230**, 120594–120626. 10.1016/j.eswa.2023.120594 (2023).

[51] Wilcoxon, F. Individual comparisons by ranking methods. *Biom. Bull.* **1**, 80–83. 10.2307/3001968 (1945).

[52] Brajević, I. et al. Hybrid sine cosine algorithm for solving engineering optimization problems. *Mathematics* **10**, 4555. 10.3390/math10234555 (2022).

[53] Hussain, K., Salleh, M. N. M., Cheng, S. & Shi, Y. On the exploration and exploitation in popular swarm-based metaheuristic algorithms. *Neural Comput. Appl.* **31**, 7665–7683. 10.1007/s00521-018-3592-0 (2019).

